# Acellular scaffold-based approach for in situ genetic engineering of host T-cells in solid tumor immunotherapy

**DOI:** 10.1186/s40779-023-00503-6

**Published:** 2024-01-04

**Authors:** Hiren Y. Dandia, Mamatha M. Pillai, Deepak Sharma, Meghna Suvarna, Neha Dalal, Ayush Madhok, Arvind Ingle, Shubhada V. Chiplunkar, Sanjeev Galande, Prakriti Tayalia

**Affiliations:** 1 Department of Biosciences and Bioengineering, Indian Institute of Technology Bombay, Powai, Mumbai, 400076 India; 2 Radiation Biology and Health Sciences Division, Bhabha Atomic Research Centre, Mumbai, 400085 India; 3 Centre of Excellence in Epigenetics, Department of Biology, Indian Institute of Science Education and Research, Pune, 411008 India; 4 Advanced Centre for Treatment, Research and Education in Cancer (ACTREC), Tata Memorial Centre, Kharghar, Mumbai, 410210 India

**Keywords:** Polyethylene glycol diacrylate, Poly-L-lysine, Lentiviruses, T-cell therapy, B16F10-OVA melanoma

## Abstract

**Background:**

Targeted T-cell therapy has emerged as a promising strategy for the treatment of hematological malignancies. However, its application to solid tumors presents significant challenges due to the limited accessibility and heterogeneity. Localized delivery of tumor-specific T-cells using biomaterials has shown promise, however, procedures required for genetic modification and generation of a sufficient number of tumor-specific T-cells ex vivo remain major obstacles due to cost and time constraints.

**Methods:**

Polyethylene glycol (PEG)-based three-dimensional (3D) scaffolds were developed and conjugated with positively charged poly-L-lysine (PLL) using carbamide chemistry for efficient loading of lentiviruses (LVs) carrying tumor antigen-specific T-cell receptors (TCRs). The physical and biological properties of the scaffold were extensively characterized. Further, the scaffold loaded with OVA-TCR LVs was implanted in B16F10 cells expressing ovalbumin (B16-OVA) tumor model to evaluate the anti-tumor response and the presence of transduced T-cells.

**Results:**

Our findings demonstrate that the scaffolds do not induce any systemic inflammation upon subcutaneous implantation and effectively recruit T-cells to the site. In B16-OVA melanoma tumor-bearing mice, the scaffolds efficiently transduce host T-cells with OVA-specific TCRs. These genetically modified T-cells exhibit homing capability towards the tumor and secondary lymphoid organs, resulting in a significant reduction of tumor size and systemic increase in anti-tumor cytokines. Immune cell profiling revealed a significantly high percentage of transduced T-cells and a notable reduction in suppressor immune cells within the tumors of mice implanted with these scaffolds.

**Conclusion:**

Our scaffold-based T-cell therapy presents an innovative in situ localized approach for programming T-cells to target solid tumors. This approach offers a viable alternative to in vitro manipulation of T-cells, circumventing the need for large-scale in vitro generation and culture of tumor-specific T-cells. It offers an off-the-shelf alternative that facilitates the use of host cells instead of allogeneic cells, thereby, overcoming a major hurdle.

**Supplementary Information:**

The online version contains supplementary material available at 10.1186/s40779-023-00503-6.

## Background

Genetic programming of T-cells for cancer immunotherapy involves isolating T-cells from patients by leukapheresis, followed by extensive labor-intensive activation, expansion and reprogramming of T-cells with genetic codes (using viral vectors or electroporation) to produce receptors on T-cells that recognize cancer cells [1]. These tumor-specific T-cells are grown in bioreactors to generate millions of cells and then reinfused into patients, where they further multiply and kill cancer cells [2, 3]. This strategy has shown significant clinical efficacy for the treatment of CD19-targeted B-cell malignancies and has received two major U.S. Food and Drug Administration approvals in August 2017 [4–7]. However, this treatment therapy, which involves extracorporeal manipulation of patient T-cells, costs about 200–400,000 dollars per patient and is long and laborious, thereby, making it infeasible for many patients despite its potential [8, 9]. These limitations have spurred research into improved manufacturing protocols, including approaches that involve the use of automated equipment to reduce labor and process variability. The development of commercial allogeneic programmed T-cells is another strategy to improve patient compliance, but allogeneic responses of the host immune system to in vitro programmed T-cells and graft-vs-host disease still need to be addressed [10, 11]. Furthermore, transient in vitro culture of T-cells can lead to terminal differentiation, which may affect their function, viability and persistence after transplantation [12, 13].

Despite the initial success of T-cell immunotherapy for hematological malignancies, reprogramming T-cells for solid tumors is still in its infancy, as systemic delivery of T-cells makes them either inaccessible to solid tumors or incapable of penetrating or surviving the immunosuppressive niche generated by solid tumors [14, 15]. Various cellular strategies involving localized delivery of tumor-specific T-cells or immunotherapeutic agents via biomaterial-based approaches [16–19] will help overcome this challenge. Specifically, a localized material-based strategy can limit the systemic side effects and access to vital organs, and also help cross the biological barrier in solid tumors for subsequent accumulation at the tumor site [20]. Biomaterials have also been employed for in vitro development of artificial antigen-presenting cells loaded with CD3, CD28 and interleukin (IL)-2 that help with the in vitro activation, proliferation and generation of a large number of T-cells [21–23]. Other cellular strategies involving co-incubating lentiviruses (LVs) and T-cells in cryogels followed by implantation of cell-loaded cryogels have shown response in hematological malignancies [24]. However, acellular strategies targeting immune cells for solid tumor immunotherapy have not yet been demonstrated. Gene delivery via lentiviral immobilization on matrices has been shown to be useful for regenerative strategies [25–27] and for delivering immunomodulatory cytokines [28], but functional studies of T-cells transduction for the anti-tumor response have not been demonstrated. Hence, a localized approach that can program the host T-cells in vivo with tumor antigen-specific receptors without in vitro manipulation would waive the need for large-scale generation and culture of tumor-specific T-cells in vitro, and would also facilitate the use of host T-cells instead of allogeneic T-cells. Biomaterial-based engineering strategies have enabled in situ programming of host T-cells via chimeric antigen receptor-loaded nanocarriers [29﻿]. However, short circulation time and systemic delivery of nanoparticles remain a concern for their application in the treatment of solid tumors. Therefore, there is a need to develop a strategy that allows in situ and localized manipulation of T-cells suitable for solid tumor therapy.

In our previous study, a three-dimensional (3D) bioactive polyethylene glycol diacrylate–poly-L-lysine (PEGDA–PLL) matrix has been demonstrated to efficiently deliver genes in vivo [30]. In this study, we demonstrate the application of this PEGDA–PLL matrix for the reduction of solid tumors via in vivo genetic programming of T-cells without the need for in vitro manipulation. This implantable bioactive 3D matrix is used to deliver B16F10 cells expressing ovalbumin (B16-OVA)-specific receptors to T-cells, leading to the generation of tumor-specific T-cells in vivo.

## Materials and methods

### Preparation and fabrication of PEGDA–PLL matrix

PEGDA matrices were modified with PLL using carbamide chemistry to increase their ability to bind LVs as previously reported [30]. The process involves dabbing matrices with 10% w/v benzophenone in ethanol, followed by 20% w/v acrylic acid, benzyl alcohol, and sodium periodate, and exposing each side to ultraviolet (UV) light for 10 min. The matrices were washed and then treated with 1-ethyl-3-(3-dimethyl aminopropyl) carbodiimide (EDC) and N-hydroxy-succinimide (NHS) solutions to activate the functional groups for PLL binding. The matrices were washed with phosphate buffered saline (PBS) and reacted with PLL (0.45 mg/ml) solution overnight at 4 °C. The matrices were washed, dried and stored at −20 °C for further use. The concentration of PLL was determined by measuring the absorbance of supernatant at 222 nm using a UV–visible spectrophotometer (Varioskan, Thermofisher, USA). The amount of PLL that gets linked with PEGDA is obtained by subtracting the unbound PLL in the supernatant from the total amount of PLL that was added.

### Physical characterization of matrices

The microstructure of scaffolds was observed using a Scanning Electron Microscope (SEM; ProX G5, The Netherlands). For this, lyophilized or freeze-dried samples were sputter-coated with gold and visualized under the microscope. Pore size was calculated using SEM images and analyzing a minimum of 100 pores using ImageJ software. Stereomicroscope (SMZ18, Nikon, Japan) was used to visualize the overall structure as well as the dry and swollen states of the fabricated matrices. Porosity of the samples was measured using liquid displacement method [31]. Briefly, 10 mm punches of matrices with approximately similar weight were used for this study. A known volume of hexane was taken in a measuring cylinder (*V*0), matrices were submerged in the liquid, and the new volume was measured as *V*1. The matrices were then removed and the volume was recorded as *V*2. Total porosity of the matrices was calculated as:

$${\text{Porosity}}\;(\% ) = (V0{-}V2)/(V1{-}V2) \times 100\%$$

For swelling ratio analysis, matrices were punched into 10 mm diameter disks and their dry weight (*W*1) was measured. Matrices were then kept in PBS for 24 h, taken out, dabbed with a tissue paper to remove excess water, following which the wet weight (*W*2) of the matrices was measured. Swelling ratio was calculated as follows:

$${\text{Swelling}}\;{\text{ratio}}\,(\% ) = (W2{-}W1)/W1 \times 100\%$$

### Biological characterization of matrices

Hemocompatibility test was performed according to American Society for Testing and Materials (ASTM) standard F756-00 (2000) and in accordance with the protocol approved by the Institute Ethics Committee (IEC) (IITB-IEC/2022/007). Briefly, matrices (6 mm in diameter) were incubated for 1 h in a 1:10 (blood:PBS) ratio of blood (10 ml) at 37 °C, after which the matrices were removed and centrifuged for 5 min at 2500 rpm. The supernatant of test samples (*ODt*) was used for optical density (*OD*) measurement at 540 nm. Similarly, the *OD* of completely lysed blood was used as a positive control (*ODpc*), and 1:10 diluted blood without any matrices was used as a negative control (*ODn*). Hemolysis (%) was calculated as follows:

$${\text{Hemolysis}}\;(\% ) = (ODt{-}ODn)/(ODpc{-}ODn) \times 100\%$$

In vitro cytocompatibility of matrices was analyzed using a direct contact assay (as per ISO 10993-5 and ISO 10993-13 part 3). Briefly, 6 mm diameter punches were UV sterilized and washed with ethanol. A known number of murine splenocytes was added to the matrices in a 96-well plate and maintained under standard cell culture conditions. After an appropriate time point (24, 48, 72, 96 h), the 3-(4,5-dimethylthiazol-2-yl)-2,5 diphenyl tetrazolium bromide (MTT) assay was performed. Control was maintained without scaffolds for cell viability comparison. Percent viability was calculated using the formula:

$${\text{Viability}}\;(\% ) = {\text{Mean}}\;OD_{{{\text{sample}}}} /{\text{Mean}}\;OD_{{{\text{control}}}} \times 100\%$$

Furthermore, 5-(and 6)-carboxyfluorescein diacetate succinimidyl ester (CFSE) labeled T-cells were cultured in the PEGDA and PEGDA–PLL matrices to study cellular retention and biocompatibility. Briefly, the 1 × 10^6^ T-cells (CFSE labeled) were added to the 6 mm matrices in minimal volume and allowed to incubate for 3 h to facilitate attachment. Following this the media was replenished and cultured for a period of 24 h before imaging. Since very few cells were seen attached to the matrices, cells were retrieved using BD cell recovery solution and trypan blue dye exclusion assay was performed to assess the viability of retained cells.

### Cell lines

B16-OVA melanoma cells were kind gift from Dr. Amit Awasthi (TSHTI, India) and were cultured in complete Roswell Park Memorial Institute (RPMI) 1640 medium (AT162, Himedia, India) with 10% heat-inactivated fetal bovine serum (FBS; RM1112, Himedia, India), 100 U/ml penicillin and 100 μg/ml streptomycin (A002, Himedia, India) and 10 mmol/L 4-(2-hydroxyethyl)-1-piperazineethanesulfonic acid (HEPES; MB016, Himedia, India). The LentiX lentiviral packaging cell line were kind gift from Dr. Rahul Purwar (IITB, India) and were cultured in Dulbecco’s modified Eagle’s medium (DMEM; AT007, Himedia, India) containing 10% FBS, 2 mmol/L glutamate (TCL030, Himedia, India), 1% non-essential amino acid (ACL006, Himedia, India), 100 U/ml penicillin and 100 μg/ml streptomycin (Himedia, India). Mice splenic T-cells were cultured in complete RPMI 1640 medium (Himedia, India) with 10% heat-inactivated FBS (Himedia, India), 2 mmol/L glutamate (Himedia, India), 1% non-essential amino acid (Himedia, India), 50 U/ml recombinant human interleukin-2 (rhIL-2; 200-02, Peprotech, USA), 100 U/ml penicillin and 100 μg/ml streptomycin (Himedia, India).

### Flow cytometry and antibodies

Monoclonal antibodies specific for mouse CD3 (Pacific Blue, HM3428, Invitrogen, USA), CD4 (APC, 17-0042-82, Invitrogen, USA), CD8a (PE, 553033, BD Biosciences, USA), OVA-TCR (APC, 17-5796-82, Invitrogen, USA), F4/80 (FITC, 123107, Biolegend, USA), CD80 (PE-Cy5, MA5-28657, Invitrogen, USA), CD206 (APC, 141708, Biolegend, USA), CD11c (APC, 17-0114-82, Invitrogen, USA), CD19 (PE, 557399, BD Biosciences, USA), CD14 (PE, 553740, BD Biosciences, USA), FOXP3 (PE, 126404, Biolegend, USA), CD62L (APC, 17-0621-82, Invitrogen, USA), CD69 (PE, 553237, BD Biosciences, USA), CD86 (PE, 12-0862-82, Invitrogen, USA), CD34 (PE, 128610, Biolegend, USA) and CD49f (APC, 313616, Biolegend, USA) were purchased, and used as per manufacturer’s instructions. Non-specific binding of all the antibodies was blocked with anti-mouse purified CD16/32 (16-0161-85, Invitrogen, USA) before staining for specific antibodies. All samples were acquired on a BD FACSAria III with BD FACS software version 10, and a minimum of 10,000 events was acquired per sample. Samples were analyzed using FlowJo software version 10.

### In vivo experiments

A total of 228 C57BL/6 wild type (WT) and 68 C57BL/6 OT-1 transgenic mice (8–12 weeks old female) were housed at Institute Animal Facilities with license No. 65/GO/ReBiBt/S/99/CPCSEA and 1496/GO/ReBi/S/11/CPCSEA respectively. For cellular infiltration, biocompatibility and bioluminescence studies, 3 animals with similar weights per group per time point were used. For anti-tumor studies, animals with similar weights were randomly grouped into BLV, SLV and no treatment groups having 8 mice per group. All animal studies were performed as per the protocols approved by the Institute Animal Ethics Committee (IAEC number 05/2020 and 2021_02/06).

#### Cell infiltration and biocompatibility studies

PEGDA or PEGDA–PLL matrices were implanted on the dorsal side of WT mice (6 mice per group) and explanted on day 3 and day 7. Cells were recovered from the scaffolds by cell recovery solution (354253, BD Bioscience, USA), following which they were stained with immune cell markers, namely, anti-mouse CD3, CD86, F4/80, CD11c, CD14 and CD19, and analyzed via flow cytometry to check for their presence within the implant. The skin around the implant was also isolated and incubated in trypsin overnight at 4 °C. The skin was then scraped to isolate the cells within the skin and stained with anti-mouse CD3, CD34 and CD49f for characterization of skin resident T-cells and stem cells, respectively. Further, inflammatory cytokine levels [mainly IL-6 and tumor necrosis factor-α (TNF-α)] from the implanted mice were performed using GeniePlex mouse cytokine analysis kit (MOAMPM015, AssayGenie, UK) as per the manufacturer’s protocol. Spleen size and weights were also analyzed to study the systemic toxicity. Furthermore, mouse blood was collected from retro-orbital plexus in an ethylenediaminetetraacetic acid-coated tubes and blood samples (20 µl) were analysed on Rayto 3 part WBC differential Veterinary Hematology Analyser (RT7600S, Rayto, China) to study the effect of implant on immune cell population.

#### Histological analysis

After subcutaneous implantation of PEGDA or PEGDA–PLL matrices (3 mice per group) on the right and left dorsal sides, these WT mice were sacrificed on day 3 and day 7. The scaffolds were explanted, embedded in paraffin, sectioned and stained with hematoxylin and eosin (HE) to evaluate the inflammatory host response as well as cellular infiltration into PEGDA and PEGDA–PLL matrices and their surrounding regions.

### LV production

Three different plasmids were used in this study for the development of second generation of lentiviral vectors. (1) pLV(Exp)-EF1α-OVA-T-cell receptor (TCR)-IRES-EGFP-WPRE-SV40 polyA (pOVA-TCR-GFP): this plasmid was cloned by https://www.vectorbuilder.com/. In this construct, OVA-TCR is expressed under the control of the elongation factor 1α (EF1α) promoter. Upstream to the EF1α promoter, the psi sequence necessary for packaging the *OT-1* gene was incorporated. To assess gene-transfer efficiency, using an internal ribosome entry site sequence we created a bicistronic genetic construct that co-expressed green fluorescent protein (GFP) along with the OVA-TCR. To increase gene expression, we placed a woodchuck hepatitis virus posttranscriptional regulatory element (WPRE) between the stop codon and the Simian virus 40 (SV40) early poly-A signal. (2) pLV(Exp)-Ubi-firefly luciferase-red fluorescent protein (RFP)-WPRE-SV40 polyA (pLuc-RFP): LVs were developed using this plasmid to help monitor in vivo programming efficiency of scaffolds using bioluminescence as an assay. (3) psPAX2 and pMD2.G: both the constructs were a kind gift from Didier Trono (Addgene plasmid # 12260 and 12259 respectively). psPAX2 codes for human immunodeficiency virus-1 gag and pol genes that express proteins required for packaging of the viruses while pMD2.G codes for vesicular stomatitis virus G envelope protein that provides broad tropism and facilitates fusion of viral envelope to that of the cell membrane.

LVs were prepared using techniques established and reported previously [30]. Briefly, LentiX cells were grown in DMEM plus 10% FBS at 37 °C and 5% CO_2_. The lentiviral packaging vectors (pMD2.G, and psPAX2) were co-transfected along with pOVA-TCR-GFP or pLuc-RFP into LentiX cells using linear polyethyleneimine (25 kD, 23966, Polysciences, USA. After 48 h of transfection, the supernatant containing viruses was collected and filtered (0.45 μm). Viruses were concentrated via ultracentrifugation and resuspended in DMEM media. The virus titer was determined by serially diluting the viruses, adding them to LentiX cells, incubating for 24 h at standard culture conditions and analyzing for GFP expression after 48 h. Infectious units (IU) of GFP expressing LVs were determined by flow cytometry by analyzing the number of cells expressing GFP after 3 d of incubation of serially diluted viruses with LentiX cells.

### Preparation of LV-immobilized PEGDA–PLL matrix

LVs encoding firefly luciferase (0.5 × 10^7^ LVs) or OVA-TCR (1 × 10^7^ LVs) were resuspended in a minimum volume (50 μl) of complete DMEM and incubated with sterilized PEGDA–PLL matrix at 4 °C for 30 min. These virus-loaded PEGDA–PLL matrices (immobilized with LVs encoding firefly luciferase or OVA-TCR were used for in vivo transduction or anti-tumor studies.

### Assessment of retention and activity of viruses immobilized on PEGDA–PLL matrix

PEGDA and PEGDA–PLL matrices were sterilized and incubated with OVA-TCR expressing LVs at different viral loads (10^6^, 10^7^, and 10^8^ LVs) for 1 h on ice. These gels were then placed in a 48-well plate and incubated with DMEM medium (containing 10% FBS) at 37 °C. Supernatant was collected and an equal amount was replenished at different time points (3, 6, 12 and 24 h). The collected media (supernatant), containing the released viruses, was added to a monolayer of LentiX cells and incubated for 2 d. The released viruses led to transduction (expression of GFP) of LentiX cells, after which cells were trypsinized and analyzed via flow cytometry to quantify GFP expression in order to assess the number of released viruses.

### In vivo transduction efficiency via bioluminescence study

To determine the in vivo efficacy of gene delivery by LVs immobilized on PEGDA–PLL matrix, 0.5 × 10^7^ firefly luciferase-expressing lentiviruses (Luc-LVs) were incubated with sterilized PEGDA–PLL matrix for 1 h at 4 °C. Animals with similar weights were segregated randomly into two experimental groups (3 animals per group), namely, bolus lentiviral delivery where bolus injection of LVs was done next to an implanted blank PEGDA–PLL matrix without LVs, and PEGDA–PLL mediated lentiviral delivery where PEGDA–PLL matrix with LVs immobilized on it was implanted. The matrices were implanted subcutaneously in 8–12 weeks old C57BL/6 mice while keeping the mice under anesthesia. In vivo luciferase expression was monitored using an in vivo imaging system (IVIS) (IVIS Spectrum, PerkinElmer, USA). For imaging, animals were injected intraperitoneally with D-luciferin (150 mg/kg body weight, 20 mg/ml in PBS; 103404-75-7, Sisco Research Laboratories Pvt. Ltd., India) using insulin syringes with 28G needles. The animals were placed in a lightproof chamber and bioluminescence images were acquired for a total of 5 min until the peak emission was confirmed. Grayscale and bioluminescence images were superimposed using the image software (Living Image, PerkinElmer, USA). A constant sized region of interest was drawn over the scaffold implantation site and at another site away from the implantation site as background. Signal intensity was monitored over time and reported as average radiance after subtracting the background. This was indicative of the luciferase expression from cells transduced by the LVs.

### Isolation of murine splenocytes

Spleen from C57BL/6 mice were dissected and minced to obtain single cell suspension after passing through a 40 μm cell strainer. Red blood cells (RBC) were lysed by adding 2 ml of RBC lysis buffer per spleen followed by incubation at 4 °C for 15 min with intermittent mixing. Plain RPMI was added to stop the lysis of RBC and splenocytes were first centrifuged and then washed with plain RPMI at 1000 rpm for 10 min each. Splenocytes were resuspended in complete RPMI media containing 10% heat inactivated FBS and used for further studies.

### T-cell enrichment using nylon wool method

The splenocyte suspension was loaded on an FBS activated nylon wool column and incubated for 1 h. The FBS activated nylon wool column was prepared by incubating pre-warmed FBS in nylon wool packed in a 5 ml syringe. Following incubation, the elute containing T-cells were collected and used for further in vitro studies [32]. For studying the retention of T-cells on the implant, one million T-cells were loaded onto the implant in 30 μl of complete RPMI and incubated for 2 h at 37 °C. After this, the T-cells were retrieved using a cell retrieval solution (354253, BD Biosciences, USA) and the number of cells retained on the scaffold was calculated by subtracting the number of retrieved cells from the total number of cells seeded initially. For activation of T-cells, the cells were cultured in CD3 (1 μg/ml, 555273, BD Biosciences, USA) coated plates along with soluble CD28 (2 μg/ml, 553295, BD Biosciences, USA) antibody for 48 h in presence of recombinant murine IL-2 (200-02, Peprotech, USA).

### In vitro T-cell transduction

Lentiviral gene transfer into murine T-cells was tested with different multiplicity of infection (MOI) for which required LVs were centrifuged at 2000×*g* with activated T-cells (1 × 10^6^) in a 24-well plate for 30 min followed by incubation at 37 °C for 6 h. After this, 1 ml of pre-warmed RPMI containing 50 U/ml rhIL-2 was added and 72 h later, T-cells were analyzed for gene expression via flow cytometry and functional in vitro assays. Viability was assessed using flow cytometry wherein cells were stained with propidium iodide (P3566, Invitrogen, USA). For lentiviral gene transfer into LentiX cells, viruses at different MOI were incubated with cells (1 × 10^6^) in 24-well plates and incubated at 37 °C for 24 h. After 24 h, the media was changed carefully to avoid detachment of cells and 72 h later, gene expression was analyzed using flow cytometry.

### Functional in vitro T-cell assays

#### Activation and proliferation of T-cells

Activation status of OVA-TCR expressing T-cells was assessed via flow cytometry by co-culturing with B16-OVA cells for 24 h and then staining the T-cells present in suspension with CD62L for naive T-cell marker and CD69 for early activated T-cell marker. Additionally, T-cells were labeled with CFSE (C34554, Invitrogen, USA) and co-cultured with B16-OVA cells for 24 h and 48 h following which they were analyzed for cellular antigen proliferation.

#### Cytotoxicity assay

In vitro cytotoxic activity of transduced T-cells was measured using a standard metabolic test (MTT assay) as previously described [33]. Briefly, tumor cells (B16F10-OVA) were seeded at 1 × 10^5^ cells per well in a 24-well plate and cultured for 24 h. OVA-TCR T-cells were added at an effector to target (E:T) ratio of 5:1. For cytotoxicity analysis, wells were washed with dulbecco phosphate buffered saline (DPBS; TS1006, Himedia, India) to remove dead cells and media traces. MTT reagent (TC191, Himedia, India) was added to the wells and allowed to incubate for 3 h at 37 °C, after which dimethyl sulfoxide (Himedia, India) was added to dissolve the formazan crystals and absorbance was measured at 570 nm. Cell viability was calculated as follows:

$${\text{Cell}}\;{\text{viability}}\;(\% ) = (OD570_{{{\text{sample}}}} {-}OD570_{{{\text{blank}}}} )/(OD570_{{{\text{control}}}} {-}OD570_{{{\text{blank}}}} ) \times 100\%$$

where blank and control were only media and un-transduced cells co-cultured with B16-OVA cells respectively.

#### Cytokine secretion assay

After 24 and 48 h of co-culture of B16-OVA cells with OVA-TCR expressing T-cells, the supernatant was collected and analyzed for the presence of interferon-γ (IFN-γ) and TNF-α secretion using GeniePlex Mouse cytokine analysis kit (MOAMPM015, Assay Genie, UK) as per the manufacturer’s protocol.

### Anti-tumor studies

Anti-tumor studies were conducted after injecting 3 × 10^6^ B16F10-OVA melanoma tumor cells on day 0 into C57BL/6 WT mice which were randomly grouped on the basis of their weights (8 mice per group). On day 1, when palpable tumors had already formed, either PEGDA–PLL implants immobilized with 10^7^ OVA-TCR LVs (referred to as scaffold mediated LV delivery or SLV) were implanted near the tumor site (in close proximity to inguinal lymph nodes) or 10^7^ LVs were subcutaneously injected next to a similarly located blank PEGDA–PLL implant (referred to as bolus LV delivery or BLV). Appropriate controls such as no tumor control injected with saline and no treatment surgical control were also kept. Mice were monitored for signs of discomfort and body weights were recorded regularly. Tumor sizes were measured once every two days until day 20 and tumor volumes were calculated as: (length × width^2^)/2 and compared with control groups. Blood was collected retro-orbitally and serum was used for cytokine analysis. On day 20, mice were euthanized and spleen, inguinal lymph nodes, implanted scaffold and tumors were isolated. Single-cell suspensions of spleen and lymph nodes were obtained by mincing the organs through a 70 μm cell mesh while those from implanted scaffolds were obtained after incubating with 1:3 dilution of cell recovery solution (354253, BD Bioscience, USA). These cells were stained with anti-mouse CD3, CD4, CD8 and OVA-TCR antibodies, and analyzed by flow cytometry. Similarly, tumors were disintegrated into 0.5 cm pieces and incubated with tissue digestion buffer (100 U/ml collagenase I and 100 μg/ml DNase in RPMI + 10% FBS) at 37 °C for 30 min followed by vigorous mixing to get single-cell suspensions that could pass through a 70 μm cell mesh. Immunophenotyping was done by staining 10^6^ cells with immune cell markers, namely, anti-mouse CD3, CD4, CD8 and OVA-TCR for the transduced T-cells; F4/80 and CD80 for M1 macrophages; F4/80 and CD206 for M2 macrophages; CD11c for dendritic cells, CD19 for B-cells and CD14 for monocytes. Serum of 4 mice from all groups was isolated by retro-orbital blood collection and lysis of RBC by resuspension in the RBC lysis buffer. It was then analyzed for the presence of Th1 and Th17 cytokines using the GeniePlex Mouse Th1 and Th17 cytokine analysis kit (MOAMPM015, Assay Genie, UK). Additionally, to eliminate the possibility that the anti-tumor effect seen is not because of the implant and the viruses; but because of the transduced T-cells, we implanted a control group of mice with GFP-expressing LVs (8 mice per group) that were immobilized on the implant. Mice were monitored for tumor size and body weight as previously mentioned and compared with control groups.

### Statistical analysis

In vitro experiments were conducted at least two times and reliably reproduced with similar effects. In vivo experiments were independently replicated at least two times with high population size. All quantitative data are represented as mean ± standard error mean (SEM) unless otherwise indicated. Violin plots show frequency distribution curves created by the kernel density method in which the middle solid line shows the median and the lower and upper dashed lines show the 25th and 75th percentiles, respectively. All statistical analyses were done using a two-tailed Student’s *t*-test, one-way ANOVA with Tukey’s test or two-way ANOVA with Sidak’s multiple comparison test analysis using GraphPad Prism version 9. *P* < 0.05 was considered to be statistically significant.

## Results

### PEGDA–PLL presents a biocompatible porous 3D matrix that facilitates cellular infiltration

**Fig. 1:**
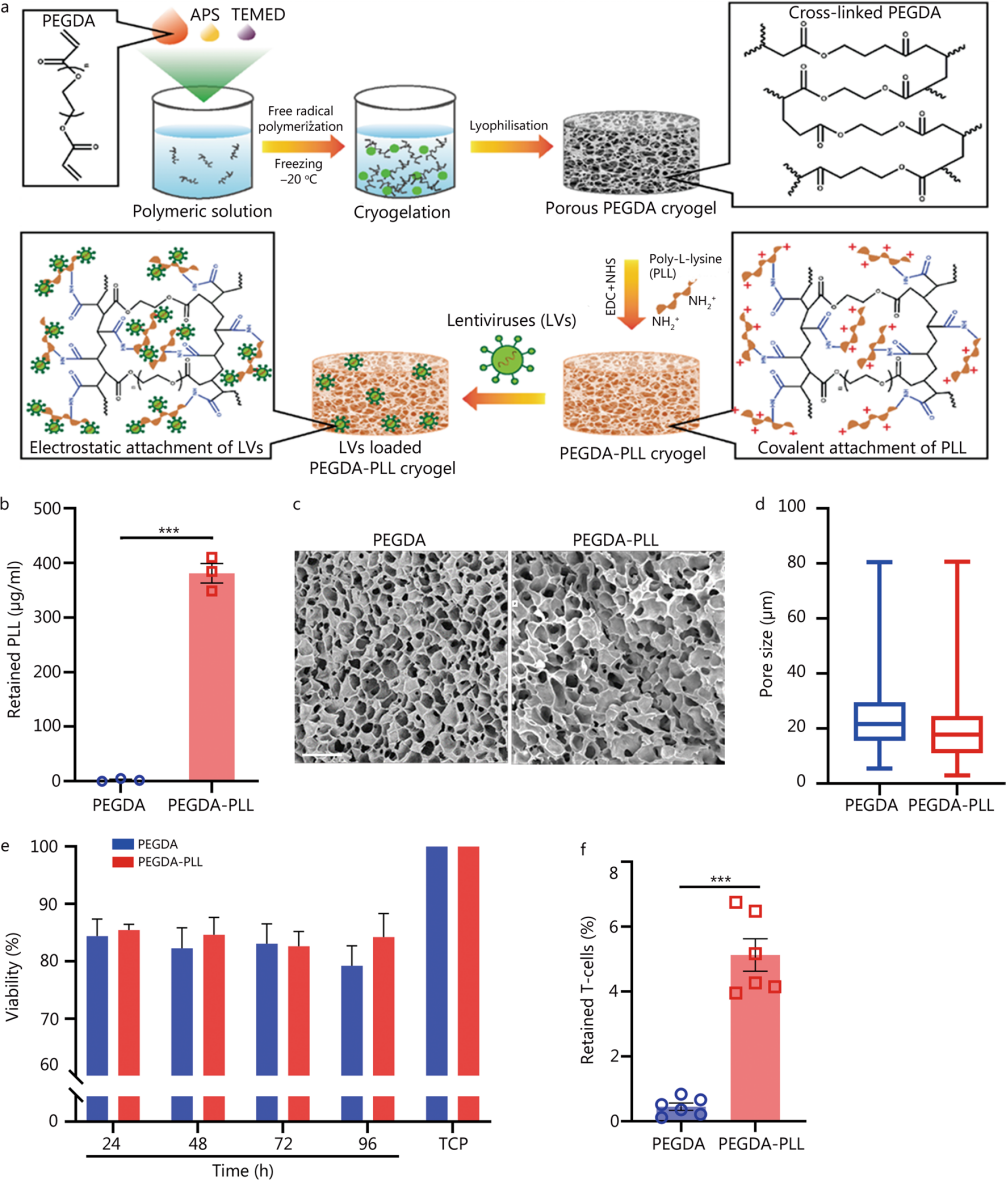
PEGDA–PLL presents a biocompatible porous 3D matrix. **a** Schematic of the preparation of bioactive PEGDA–PLL matrix. **b** Quantification of PLL retention on the matrix. **c** Scanning electron microscopy (SEM) images of PEGDA and PEGDA–PLL matrices showing macroporous structure throughout the scaffold. Scale bar = 100 µm. **d** Pore size of matrices measured using ImageJ with at least 100 pores from 3 representative images. **e** Viability of splenocytes cultured in these matrices measured at different times using MTT assay. **f** Retention of T-cells in these matrices after 24 h of culture measured using total viable cell count assay. ^***^P < 0.001, one-way ANOVA with Tukey’s test or Student’s *t*-test. PEGDA polyethylene glycol diacrylate, PLL poly-L-lysine, 3D three dimensional, APS ammonium persulphate, TEMED N,N,N′,N′-tetramethylethylenediamine, MTT 3-(4,5-dimethylthiazol-2-yl)-2,5 diphenyl tetrazolium bromide, EDC 1-ethyl-3-(3-dimethyl aminopropyl) carbodiimide, NHS N-hydroxy-succinimide, TCP tissue culture plate

PEGDA scaffolds have been used to deliver drugs and a variety of immunotherapeutic agents [30, 34–36]. Here, PEGDA was used as a material for 3D matrix due to its inertness and biocompatibility. During the preparation process, PEGDA is chemically modified and thus can be easily conjugated with positively charged PLL. As shown in Fig. 1a, the negatively charged LVs immobilize on these matrices via electrostatic interaction with the positively charged PLL. The retention of PLL on the PEGDA scaffolds was quantified as shown in Fig. 1b. It was observed that the PEGDA scaffold was capable of retaining approximately 400 μg/ml of PLL after surface modification using EDC-NHS chemistry. Scanning electron microscopy (SEM) of both the matrices (PEGDA and PEGDA–PLL) (Fig. 1c) revealed a well-connected porous structure with pores ranging from 5 to 80 µm (Fig. 1d) and a porosity of (60.7 ± 4.1)% (Additional file 1: Fig. S1a). This is required for the infiltration of host cells into these matrices in large numbers. Stereomicroscopy images of matrices in dry and swollen states and their quantification (Additional file 1: Fig. S1b) indicate their ability to swell and facilitate nutrient and cellular uptake. Furthermore, PLL modification did not alter the physical properties of matrices. When primary mouse splenocytes were cultured on these scaffolds, there was no significant change in the viability of these cells up to 96 h (*P* > 0.05, Fig. 1e). Also, when incubated with primary human blood, minimal hemolysis (2.5 ± 1.4)% was observed in the PEGDA–PLL group and was well within the acceptable limit of less than 5% specified by medical standards (Additional file 1: Fig. S1c) [37]. This supports the overall biocompatibility of the PEGDA–PLL matrix. When enriched murine T-cells were cultured on these matrices, it was found that PEGDA and PEGDA–PLL showed minimal cell retention (less than 6%), which was quantified by trypan blue dye exclusion assay (Fig. 1f). PEGDA–PLL was able to retain significantly higher cells as compared to PEGDA implant due to the positive charge of PLL. These results as visualized by confocal microscopy, confirmed no effect on the viability of cultured T-cells as shown in Additional file 1: Fig. S1d, e. Macroporous properties (Fig. 1c, Additional file 1: Fig. S1d) of PEGDA/PEGDA–PLL matrices allow cell recruitment while the absence of cell-adhesion moieties enable low cell retention (Fig. 1f), suggesting that the cells recruited into the implant are not immobilized.

**Fig. 2:**
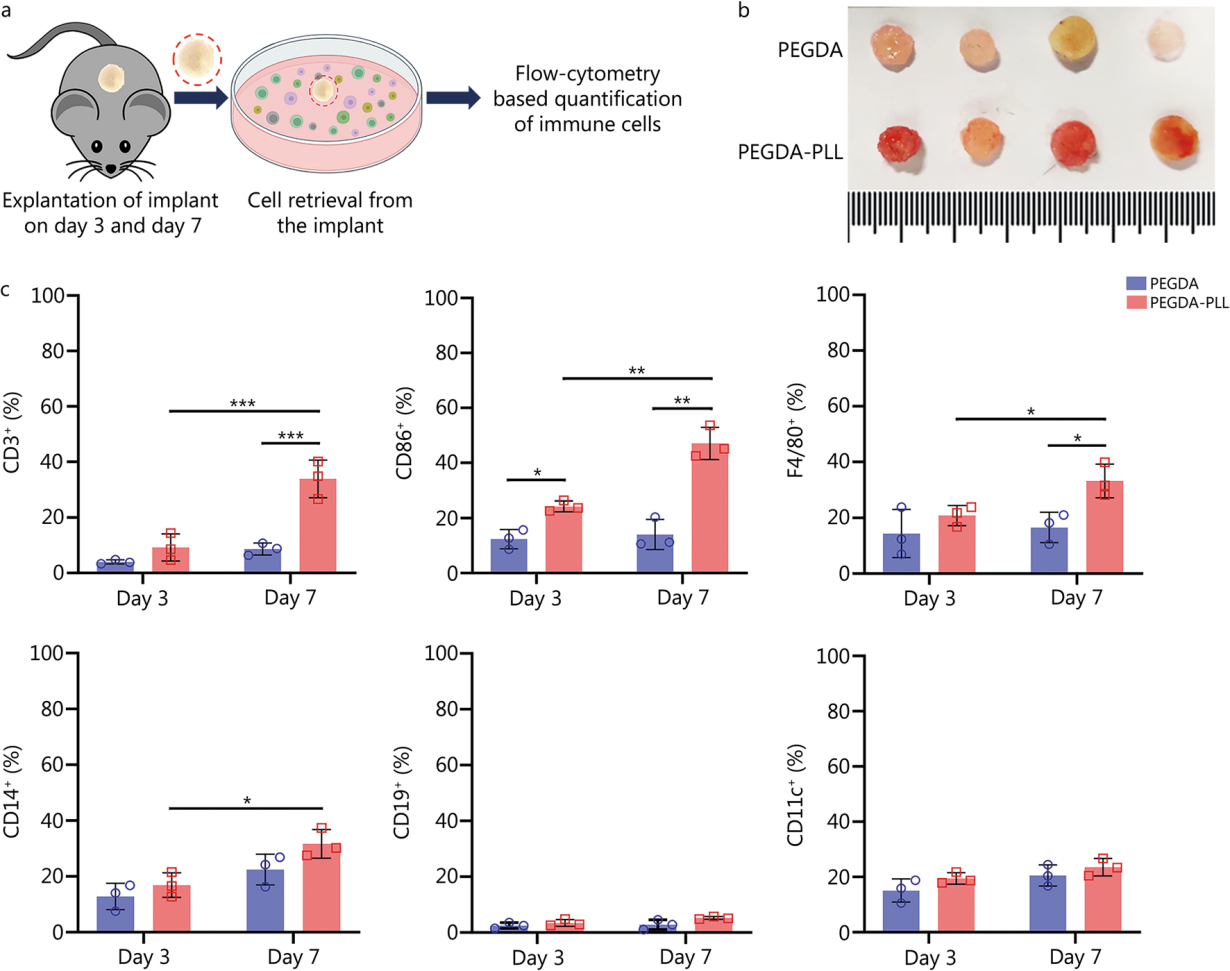
PEGDA–PLL matrix promotes more cellular infiltration. **a** Experimental design for cellular infiltration studies in 3D implants. **b** Digital micrographs of explanted implants on day 7 showing red color due to infiltration of blood vessels. **c** Quantification of different immune cells (CD3^+^, CD86^+^, F4/80^+^, CD14^+^, CD19^+^, and CD11c^+^ respectively) infiltrating the PEGDA and PEGDA–PLL implants on day 3 and day 7. ^*^P < 0.05, ^**^P < 0.01, ^***^P < 0.001, one-way ANOVA with Tukey’s test. PEGDA polyethylene glycol diacrylate, PLL poly-L-lysine

These matrices were implanted subcutaneously into the dorsal side of mice (Additional file 1: Fig. S2a) and retrieved on days 3 and 7 for subsequent analysis (Fig. 2a). No inflammation, redness or swelling associated with surgery or with the implant material was found on gross examination. On day 7, at the time of removal, the PEGDA–PLL implant was found to be red in color suggesting a greater degree of angiogenesis and cellular infiltration into the scaffold than the PEGDA implant (Fig. 2b) [25]. In addition, histological studies qualitatively confirmed that PEGDA–PLL implants had better cellular infiltration than PEGDA implants as deduced by the high cytoplasmic content observed in the H and E images, especially on day 7 (Additional file 1: Fig. S2b). Quantitative analysis showed that the cell counts of PEGDA–PLL implants were higher than those of PEGDA implants on days 3 and 7 (*P* < 0.05 or *P* < 0.01, Additional file 1: Fig. S2c). After immunophenotyping of the cells infiltrating the scaffolds on day 3 (Fig. 2c), no significant difference in the CD3^+^ population (T-cells) was observed between PEGDA and PEGDA–PLL implants. However, on day 7, the percentage of these cells was higher in the PEGDA–PLL system (about 33%) compared with the PEGDA (about 9%) system (*P* < 0.001). A thorough examination of the literature revealed that PLL, through its electrostatic polymer-receptor interactions, has the ability to induce T-cell activation by calcium signaling and altering the dynamics of TCRs [38]. Since downstream signaling leads to subsequent cytokine production, it might have triggered a chemokine directed migration of other T-cells leading to an increase in the number of T-cells infiltrating the PEGDA–PLL implant. Antigen-presenting (CD86^+^) cells were also higher in PEGDA–PLL compared with PEGDA matrix on days 3 and 7 as shown in Fig. 2c. Further characterization revealed an increase in F4/80^+^ (macrophages; Fig. 2c) on day 7. However, no significant increase in CD14^+^ (monocytes), B cell (CD19^+^) or dendritic cell (CD11c^+^) population was observed between PEGDA and PEGDA–PLL at either of the time points (Fig. 2c), although differences were observed between days 3 and 7 in the CD14^+^ (monocytes) population of the PEGDA–PLL group. Thus, from our data, we conclude that PEGDA–PLL matrix shows a better overall infiltration of immune cells, with a clear increase in T-cells. However, detailed mechanistic studies will be required to establish the role of PLL on various immune cell types.

Then, the effect of implants on peripheral tissue was evaluated (Additional file 1: Fig. S3a); it was found that PEGDA–PLL not only increased the infiltration of CD3^+^ cells into the implants (as mentioned above) but also increased the number of skin resident T-cells at day 7 (Additional file 1: Fig. S3b). However, no significant differences in the number of CD34^+^CD49f^+^ skin stem cells (Additional file 1: Fig. S3c) were observed, suggesting the lack of any local side effects, thereby confirming the compatibility of the implants. Systemic toxicity was assessed by quantifying the inflammatory cytokines in blood at day 7, and no significant differences in IL-6 and TNF-α levels were observed amongst surgical control, PEGDA and PEGDA–PLL groups (Additional file 1: Fig. S3d). Furthermore, spleen size and weight of mice did not show any significant difference between the groups (Additional file 1: Fig. S3e). Similarly, the blood cell markers (Additional file 1: Table S1) were similar in the experimental groups. This further validates the biocompatibility of the implants for in vivo application.

### PEGDA–PLL matrix facilitates LV immobilization for efficient gene delivery in vivo

**Fig. 3:**
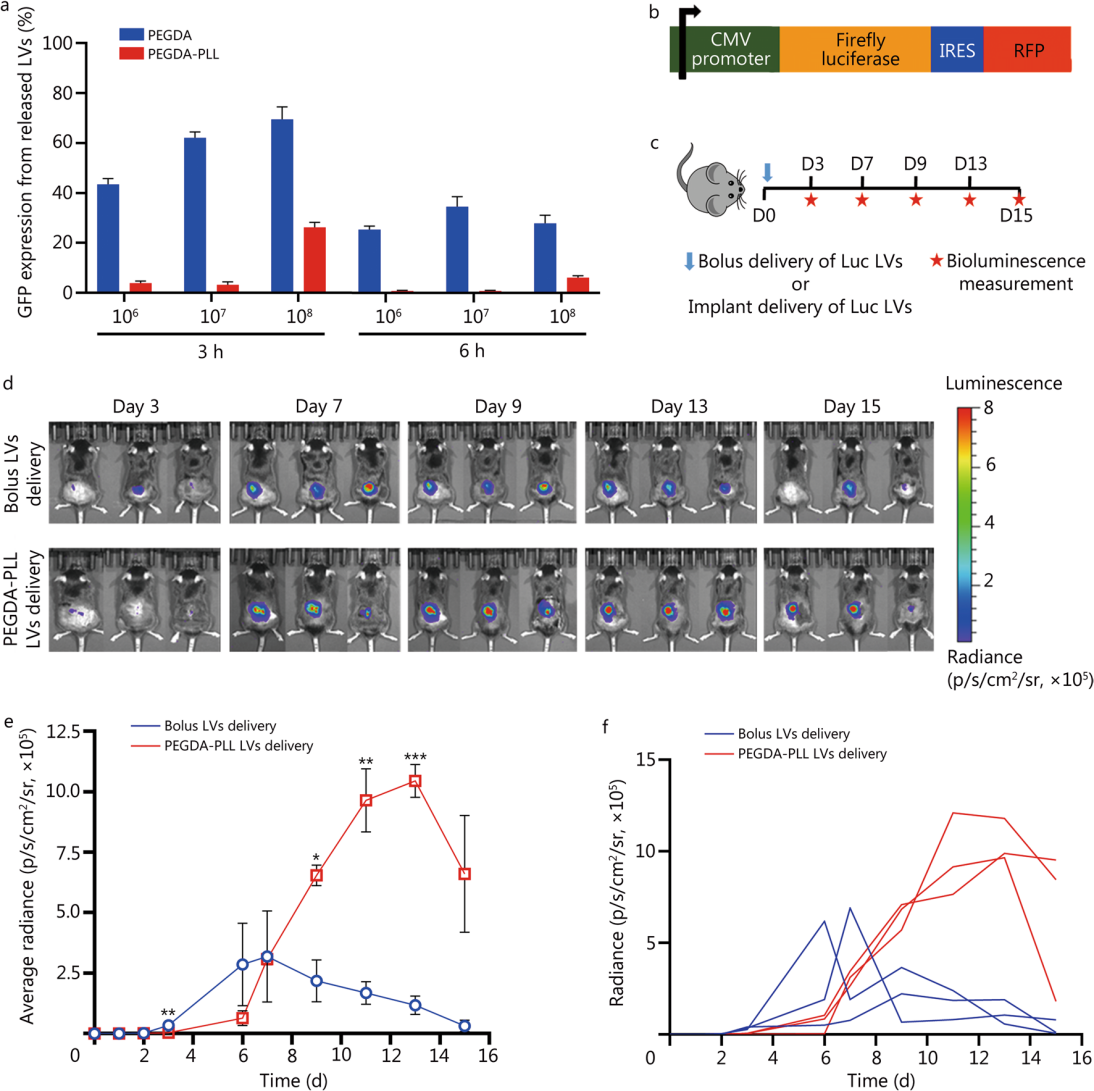
PEGDA–PLL matrix efficiently immobilizes lentiviruses (LVs) and delivers genes in vivo.** a** Percentage of GFP expression in LentiX cells when incubated for 72 h with LVs released at 3 h and 6 h from matrices immobilized with different numbers of lentiviral particles (10^6^, 10^7^, and 10^8^). **b** Schematic of Luc-RFP plasmid construct used to produce luciferase encoding LVs. **c** Experimental design of bioluminescence study conducted to visualize temporal expression of luciferase gene by delivering Luc-LVs either in a bolus manner (subcutaneous injection) or via immobilization on PEGDA–PLL implant. **d** Representative in vivo bioluminescence imaging of 3 mice per group shown after injecting D-luciferin 150 mg/kg body weight of mice. **e** Average radiance measured using constant-size regions of interest in mice injected with bolus LVs or implanted with PEGDA–PLL matrices immobilized with LVs. **f** Representative radiance of 3 mice per group is shown. Each line represents one animal and each point reflects the radiance captured at a particular time. ^*^P < 0.05, ^**^P < 0.01, ^***^P < 0.001 vs. bolus LVs delivery, Student’s *t*-test. GFP green fluorescent protein PEGDA polyethylene glycol diacrylate, PLL poly-L-lysine, CMV cytomegalovirus, IRES internal ribosome entry sites, RFP red fluorescent protein, Luc lucierfase

LVs, the vectors that carry the gene of interest and confer genetic modification on cells, are inherently immunogenic but are neutralized by complement activation during their systemic administration, resulting in low availability [39]. Hence, to efficiently transduce T-cells, LVs must be loaded and immobilized to a matrix that acts as a gene reservoir and also protects the virus from immune inactivation [40]. As shown in Fig. 3a, unbound GFP-expressing LVs (GFP-LVs) were released from PEGDA matrix, while minimal release occurred from the PEGDA–PLL matrix within the first 3 to 6 h. There was little release at 12 or 24 h (Additional file 1: Fig. S4a) from either of these matrices. Thus, it was confirmed the PEGDA–PLL matrix retained the viruses more efficiently than the PEGDA matrix. However, when increasing titers of GFP-LVs were incubated with these matrices, PEGDA–PLL matrix was found to retain the most when incubated with 10^6^ and 10^7^ LVs. When the number of LVs was increased to 10^8^, a large amount of LVs was released during the first 3 h indicating the saturation of viral load on the matrix. No viruses were observed to be released from either PEGDA or PEGDA–PLL matrices between 12 and 24 h (Additional file 1: Fig. S4a), suggesting that none of these matrices had any remnant viruses waiting to be released at later time points. This further indicates that viruses were successfully immobilized on the PEGDA–PLL matrix and that their loading threshold was reached at 10^8^ viral particles. Therefore, PEGDA–PLL matrix loaded with 10^7^ LVs was used for all subsequent in vivo experiments.

In addition, we employed a firefly luciferase-RFP plasmid under cytomegalovirus promoter (Fig. 3b) to generate LVs. These were used to evaluate their function when immobilized on the PEGDA–PLL matrix and investigate the temporal effect of scaffold-mediated gene delivery in vivo. The Luc-LVs immobilized on the scaffolds were subcutaneously implanted into C57BL/6 mice. Using IVIS imaging, bioluminescence was measured for 15 d to quantify luciferase expression. As depicted schematically in Fig. 3c, bolus subcutaneous administration of these LVs (without immobilization on implants) was used as a control. At 3 d, low level of transduction was found in mice injected with bolus LVs as seen by bioluminescence and luciferase expression (Fig. 3d). Conversely, PEGDA–PLL-mediated LVs delivery did not show any bioluminescence in the first 3 d, but exhibited significantly higher intensity between 7 and 15 d when compared to the bolus LVs delivery counterpart (Fig. 3e, f). Bolus LVs delivery seems to suggest that the LVs may be undergoing a phenomenon similar to immune neutralization which occurs with systemic delivery of LVs [39, 40]. Our bioluminescence data indicate that the PEGDA–PLL implants may be protecting the LV activity from this phenomenon. Thus, despite a lag, more sustained and efficient in vivo transduction was achieved with PEGDA–PLL implants compared to bolus administration.

### In vitro transduction with OVA-TCR LVs generates functional tumor-specific T-cells

**Fig. 4:**
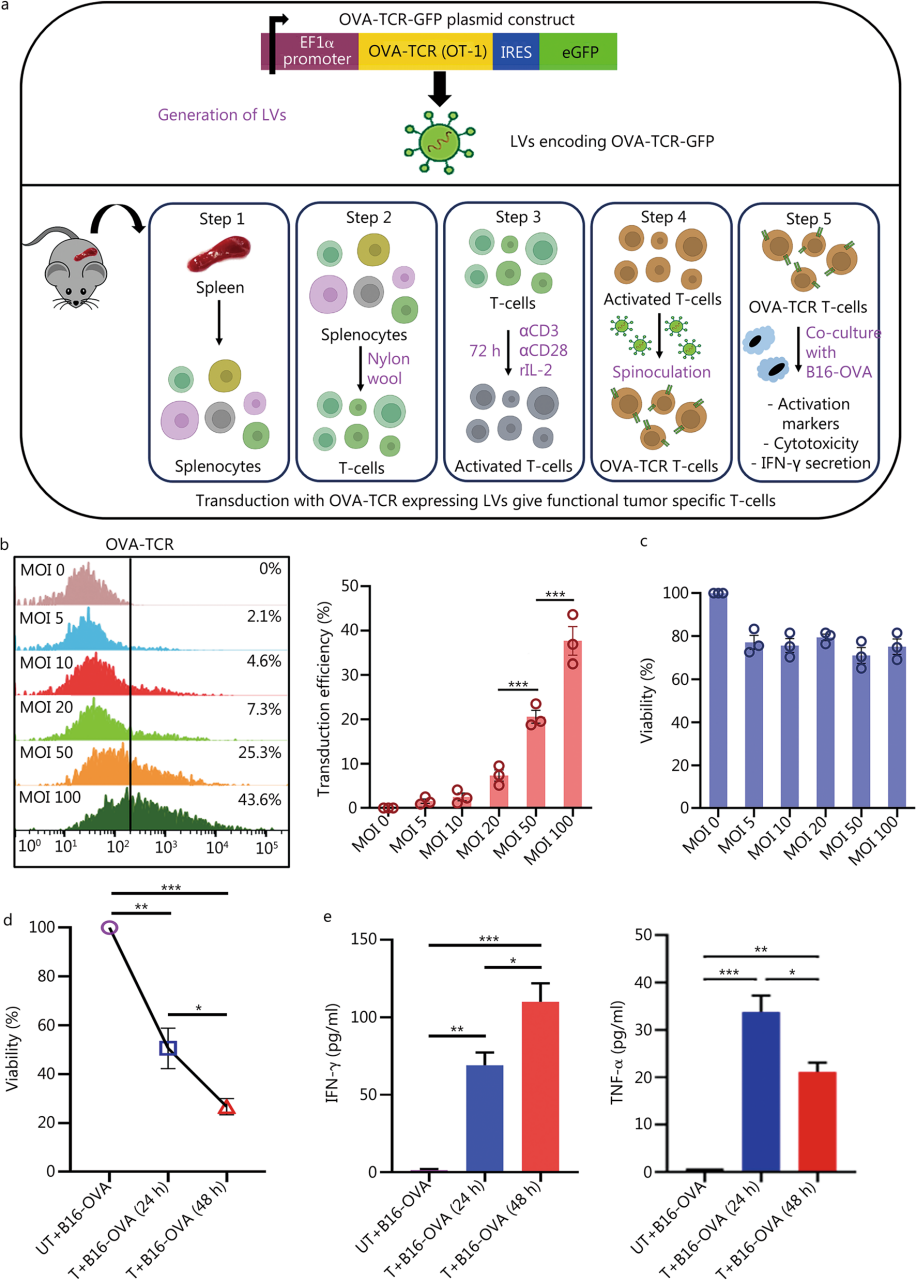
OVA-TCR lentiviruses (LVs) facilitate gene transfer in primary T-cells and provide anti-tumor effects in vitro.** a** Overview of the process of LVs mediated transduction of murine spleen T-cells with OVA-TCR GFP. Top panel shows the plasmid construct used for transgene expression. Bottom panel shows the overall procedure of T-cell transduction for functional studies. **b** Overlaid flow cytometry histogram plots and quantification of T-cell transduction depicting OVA-TCR expression in T-cells transduced via LVs at varying multiplicity of infection (MOI). **c** Quantification of T-cell viability when subjected to transduction at different MOI. **d** Percentage of B16-OVA tumor cells viability when co-cultured with non-transduced T-cells (UT + B16-OVA) and OVA-TCR expressing transduced T-cells (T + B16-OVA) at 24 h and 48 h at an effector to target ratio of 5:1. **e** Quantification of IFN-γ and TNF-α released (as assessed by ELISA) in the supernatant of co-culture of T-cells with tumor cells. ^*^P < 0.05, ^**^P < 0.01, ^***^P < 0.001, one-way ANOVA with Tukey’s test. OVA-TCR ovalbumin T-cell receptor, GFP green fluorescent protein, EF1α elongation factor 1α, IRES internal ribosome entry sites, eGFP enhanced green fluorescent protein, αCD3 cluster of differentiation 3, αCD28 cluster of differentiation 28, rIL-2 recombinant interleukin-2, IFN-γ interferon-γ, TNF-α tumor necrosis factor-α

In this study, LVs were chosen as transduction vectors because they can efficiently transduce both actively dividing and quiescent T-cells. They were generated by constructing plasmid expressing OVA-TCR under the EF1α promoter (Fig. 4a), as EF1α is known to be resistant to epigenetic modifications [41, 42]. EF1α has more binding sites for transcription factors expressed in T-cells than other promoters [43]. The infectivity of LVs was assessed by incubating the viral supernatant with LentiX cells and quantifying the reporter gene expression in these cells (Additional file 1: Fig. S4b). LVs were incubated with LentiX cells at different ratios and it was found that transfection efficiency (or infectivity) increases with the number of LVs per cell (referred to as MOI) (Additional file 1: Fig. S4c). LVs were spinoculated with activated mouse spleen T-cells at different MOIs and OVA-TCR expression on these cells was analyzed after 72 h of incubation. Highest transduction efficiency (approximately 32%) was observed at an MOI of 100 (Fig. 4b). The viability of transduced cells was above 70% for both LentiX cells (Additional file 1: Fig. S4d) and T-cells (Fig. 4c) across all MOIs. To assess the effect of in vitro transduction on functionality of T-cells, control (untransduced) and transduced T-cells expressing OVA-TCR were co-cultured with B16-OVA tumor cells at an effector-to-target (E:T) ratio of 5:1 as per literature [33]. Transduced T-cells exhibited high expression level of CD69 (an activation marker) but low expression level of CD62L (a naive T-cell marker) compared to untransduced T-cells (Additional file 1: Fig. S4e). At 24 h and 48 h, untransduced T cells failed to eradicate tumor cells, while the transduced OVA-TCR expressing T-cells showed significant cytotoxicity against B16-OVA cells (Fig. 4d) and secreted elevated levels of effector cytokines such as IFN-γ and TNF-α (Fig. 4e). These cytokines showed a substantial increase at 24 h; however, TNF-α level slightly declined at 48 h. Study by Brehm et al. [44] has demonstrated that TNF-α is one of the cytokines released within the first few hours following antigen encounter. Upon TCR binding to the antigen, rapid release of TNF-α occurs and leads to initiation of immune response. Subsequently, effective CD8^+^ T cell-mediated response requires IFN-γ as well, however, Ye et al. [45] have shown that production of TNF-α by CD8^+^ T-cells does not always coincide with INF-γ production. Additionally, when CFSE-labeled OVA-TCR expressing T-cells were co-cultured with B16-OVA cells, a notable increase in proliferation (due to clonal expansion) of T-cells was observed in response to OVA antigen expressed on tumor cells (Additional file 1: Fig. S4f). Thus, these experiments validate both the design of OVA-TCR plasmid and the functionality of LV-transduced T-cells generated using this plasmid.

### PEGDA–PLL implants enable host T-cells to mount a functional anti-tumor response in vivo

**Fig. 5:**
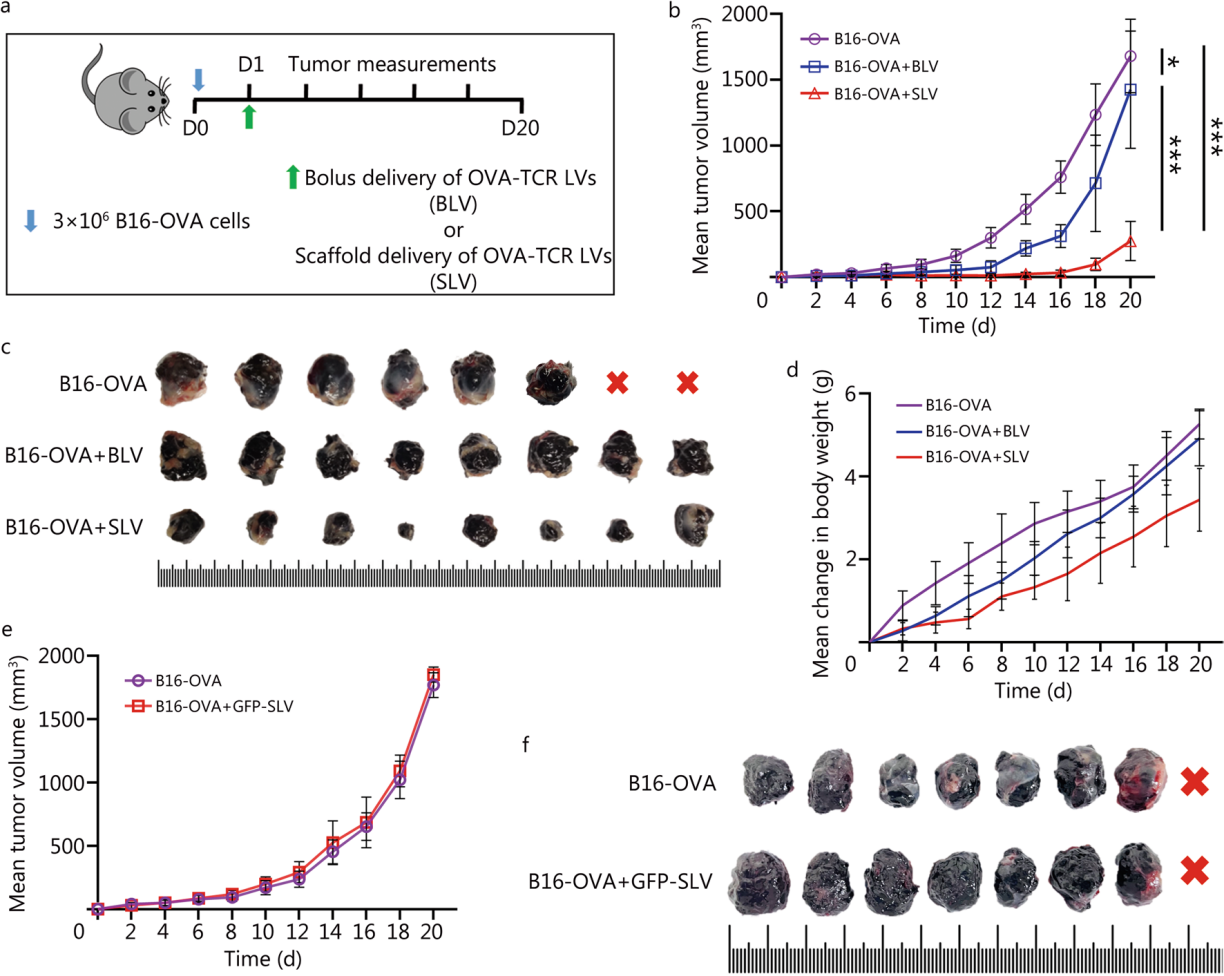
PEGDA–PLL implants program host T-cells leading to reduction of tumor size in vivo.** a** Experimental design for anti-tumor study conducted in C57BL/6 mice bearing B16-OVA melanoma tumor, with bolus (B16-OVA + BLV) or PEGDA–PLL (B16-OVA + SLV) matrices immobilized with OVA-TCR encoding lentiviruses (LVs). **b** Mean tumor volume measured for treatment as well as the untreated (B16-OVA) groups. **c** Comparison of tumors explanted on day 20 from various treatment groups. **d** Change in body weight for each treatment group. **e** Mean tumor volume measured for GFP expressing lentivirus loaded implant (GFP-SLV) as well as the untreated (B16-OVA) groups. **f** Comparison of tumors explanted on day 20 from various treatment groups. Red cross indicated that the corresponding animal died before the time-point was reached. Minimum unit of scale is 1 mm. ^*^P < 0.05, ^**^P < 0.01, ^***^P < 0.001, one-way ANOVA with Tukey’s test. PEGDA–PLL polyethylene glycol diacrylate poly-L-lysine OVA-TCR ovalbumin T-cell receptor, BLV bolus lentiviruses, SLV scaffold loaded lentiviruses, GFP green fluorescent protein

The anti-tumor activity effected by PEGDA–PLL-mediated delivery of OVA-TCR LVs was assessed after confirming successful LV transduction observed through bioluminescence experiments and suitable design of plasmid construct validated via in vitro transduction experiments. We examined the ability of the implant to transduce T-cells with OVA-TCR gene and their ability to elicit anti-tumor activity in a murine B16-OVA melanoma tumor model, as shown in Fig. 5a and Additional file 1: Fig. S5. Tumors grew rapidly in mice that were not treated with lentiviral therapy (B16-OVA) or given bolus LVs (B16-OVA + BLV), whereas mice implanted with PEGDA–PLL implants immobilized with LVs (B16-OVA + SLV) significantly controlled tumor progression (Fig. 5b, c). There was no significant change in body weight (Fig. 5d) throughout the study. In addition, to eliminate the possibility of tumor reduction due to the immunogenicity of the lentiviral vector, an additional experiment was performed in which the scaffolds were loaded with GFP LVs instead of OVA-TCR LVs and implanted into the B16-OVA tumor-bearing mice. Tumor growth observed in the GFP LVs group was similar to that observed in the no treatment control group (Fig. 5e, f). The presence of blank PEGDA–PLL scaffold in the bolus delivery (BLV) group already suggested that this effect was not due to the scaffold itself (absence of LVs) but was primarily due to the anti-tumor effect generated by OVA-TCR expressing T-cells transduced via LVs loaded on scaffold (SLV) (Fig. 5b). This confirmed that the anti-tumor effects were not due to any immunostimulatory effect caused by either the scaffold or the lentiviral vector, but stemmed from a functional aspect associated with transduced OVA-TCR expressing T-cells.

**Fig. 6:**
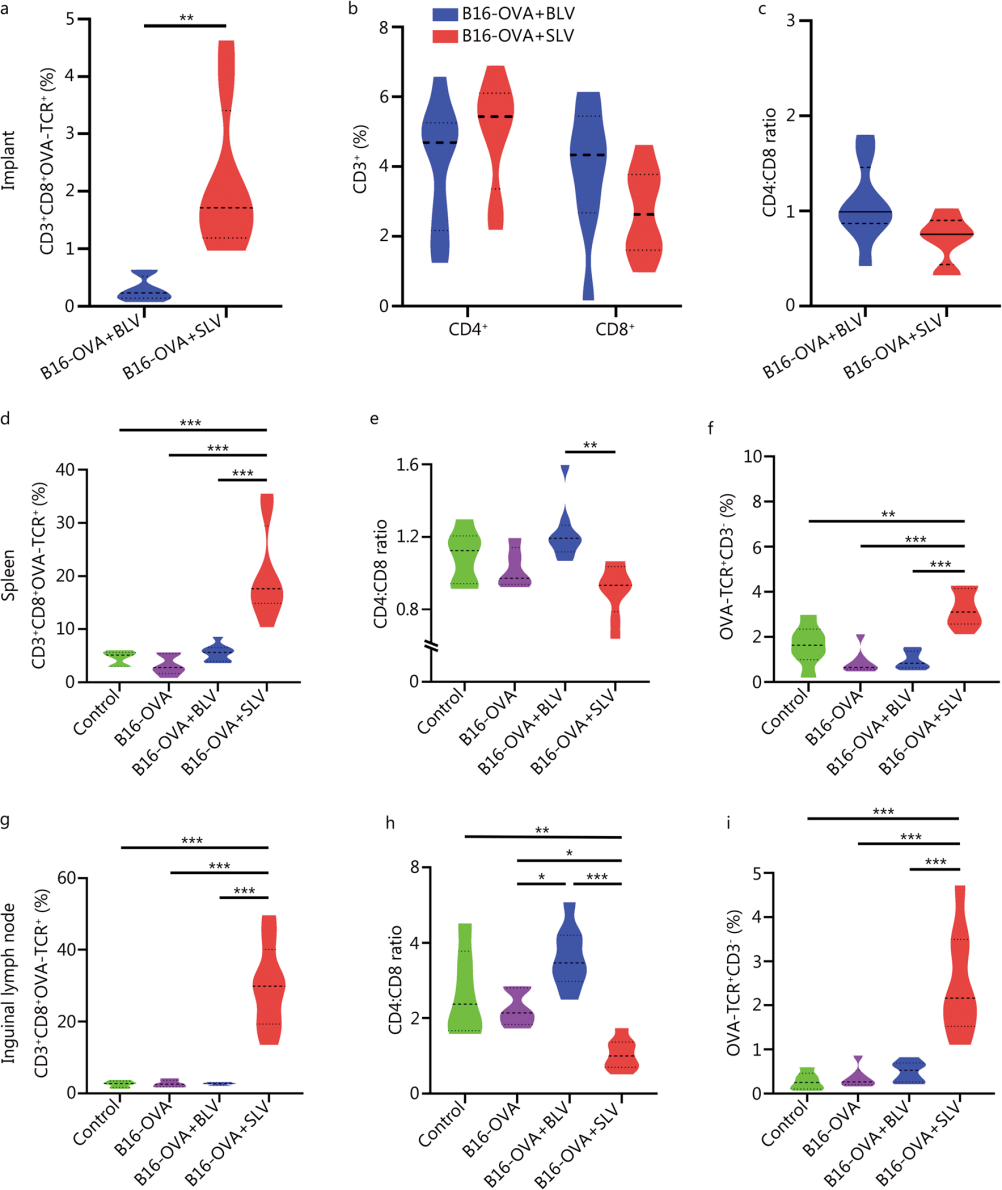
Host T-cells programmed in situ via PEGDA–PLL implants show higher infiltration into the implant and into the secondary lymphoid organs. Flow cytometry based quantification of percentage of CD3^+^CD8^+^OVA-TCR^+^ cells (**a**), CD3^+^CD4^+^ and CD3^+^CD8^+^ (**b**), CD8:CD4 ratio (**c**) in the explanted scaffolds for bolus and scaffold mediated LV delivery. Quantification of percentage of CD3^+^CD8^+^OVA-TCR^+^ cells, CD4:CD8 ratio and OVA-TCR^+^CD3^−^ cells in spleen (**d–f**) and inguinal lymph nodes (**g–i**). ^*^P < 0.05, ^**^P < 0.01, ^***^P < 0.001, one-way ANOVA with Tukey’s test. PEGDA–PLL polyethylene glycol diacrylate poly-L-lysine, OVA ovalbumin, TCR T-cell receptor, BLV bolus lentiviruses, SLV scaffold loaded lentiviruses

To examine the successful generation of OVA-TCR expressing T-cells in vivo, scaffolds were removed from mice on day 20 and various cell populations were analyzed. Compared to blank implants in BLV (bolus LVs), the number of transduced T-cells (CD3^+^CD8^+^OVA-TCR^+^ cells) in SLV implants was significantly higher (*P* < 0.001, Fig. 6a), providing evidence for the scaffold’s ability to recruit and activate cytotoxic T-cells. However, there were no significant differences observed in the percentage of CD3^+^CD4^+^ and CD3^+^CD8^+^ cells or CD4:CD8 ratio (CD3^+^CD4^+^/CD3^+^CD8^+^) found in the implants of BLV and SLV groups (Fig. 6b, c), indicating no preferential recruitment of specific T-cell subtype. Additionally, spleen and inguinal lymph nodes were collected on day 20 to examine the migration of transduced host T-cells from the implant to lymphoid organs. Spleen size and weight showed a significant increase in the SLV group when compared with other control conditions (*P* < 0.05, Additional file 1: Fig. S6). Increased spleen size is typically correlated with an enhanced immune response and may be attributed to activation and proliferation of OVA-TCR expressing T-cells generated in vivo. Therefore, using the gating strategy described in Additional file 1: Fig. S7, we analyzed various T-cell populations in the collected spleens. Higher percentages of OVA-TCR expressing T-cells, specifically CD3^+^CD8^+^OVA-TCR^+^ cells (Fig. 6d, Additional file 1: Fig. S8a) and CD3^+^CD8^−^OVA-TCR^+^ cells (Additional file 1: Fig. S8b), were found in the spleen of SLV group compared to other controls. Additionally, the percentage of CD3^+^CD8^+^ was higher than that of CD3^+^CD4^+^ cells (Fig. 6e, Additional file 1: Fig. S8c), indicating that a large number of cytotoxic T-cells (CD8^+^) induced a strong anti-tumor response. We also observed a small population of non T-cell (CD3^−^) lymphocytes expressing OVA-TCR in the spleens, although it was higher in the SLV group than other experimental groups. These OVA-TCR^+^CD3^−^ (Fig. 6f) cells may not lead to any significant response due to lack of co-stimulatory signals found only on CD3^+^ T-cells. Likewise, analysis of CD3^+^CD8^+^OVA-TCR^+^, CD3^+^CD8^−^OVA-TCR^+^, CD3^+^CD4^+^, CD3^+^CD8^+^ and OVA-TCR^+^CD3^−^ cell populations and CD4:CD8 ratio was found to be similar in the collected inguinal lymph nodes as well (Fig. 6g-i, Additional file 1: Fig. S8d-f). This indicates a higher number of effector T-cells, especially transduced (OVA-TCR^+^) T-cells in secondary lymphoid organs (spleen and lymph nodes) under SLV condition, suggesting that transduced T-cells migrate from implantation site and likely proliferate to control tumor progression. Although no mechanistic studies involving chemokines responsible for migration were performed in this study, reports suggest that in solid tumors chemokines such as CXCR3 (C-X-C motif chemokine receptor), CXCR4, CXCL12 (chemokine C-X-C motif ligand), CCL21 (chemokine ligand), and CCL19 primarily promote T-cell homing [46]. We also examined the non-specific transduction of monocyte and granulocyte population (OVA-TCR^+^) in spleen (Additional file 1: Fig. S9a), and observed no significant differences between the experimental groups and only a small percentage of transduced cells migrating to the spleen (Additional file 1: Fig. S9b). Non-specific transduction can be minimized in our system by using T-cell specific promoters that can restrict the gene expression to T-cells. Additional modifications to the implant may involve immobilizing anti-CD3/anti-CD28 antibodies or incorporation of chemokines, which could enhance the efficacy of this approach.

### Host T-cells programmed by PEGDA–PLL implants effectively infiltrate tumor and effect enhanced Th1 and Th17 responses

**Fig. 7:**
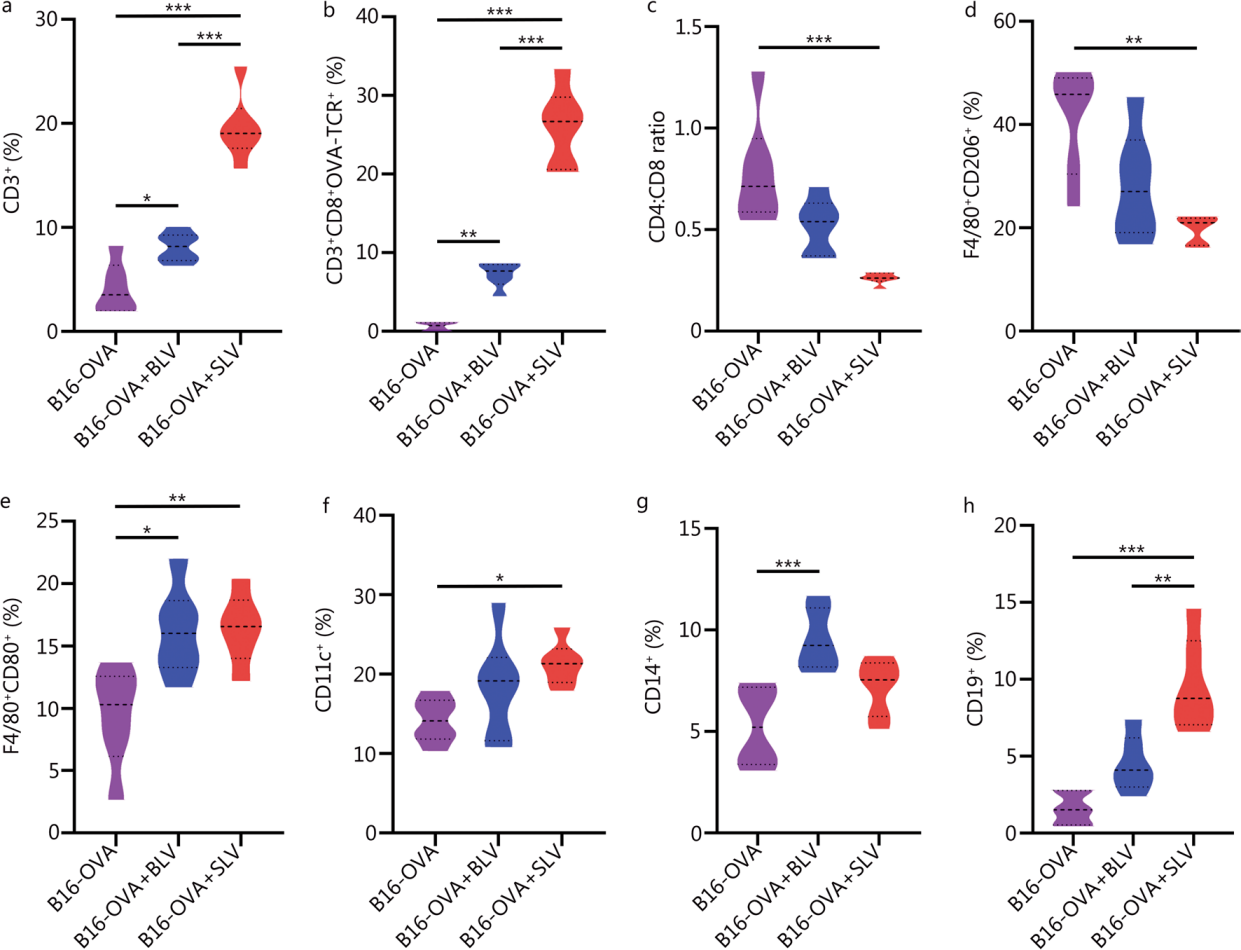
Flow cytometry-based immunoprofiling and quantification of immune cells in tumors. Cells infiltrating the tumors, representing percentage of CD3^+^ cells (**a**), CD3^+^CD8^+^OVA-TCR^+^ cells (**b**), CD4:CD8 ratio (**c**), F4/80^+^CD206^+^ M2 macrophages (**d**), F4/80^+^CD80^+^ M1 macrophages (**e**), CD11c^+^ dendritic cells (**f**), CD14^+^ monocytes (**g**), and CD19^+^ B-cells (**h**). ^*^P < 0.05, ^**^P < 0.01, ^***^P < 0.001, one-way ANOVA with Tukey’s test. OVA ovalbumin, TCR T-cell receptor, BLV bolus lentiviruses, SLV scaffold loaded lentiviruses

**Fig. 8:**
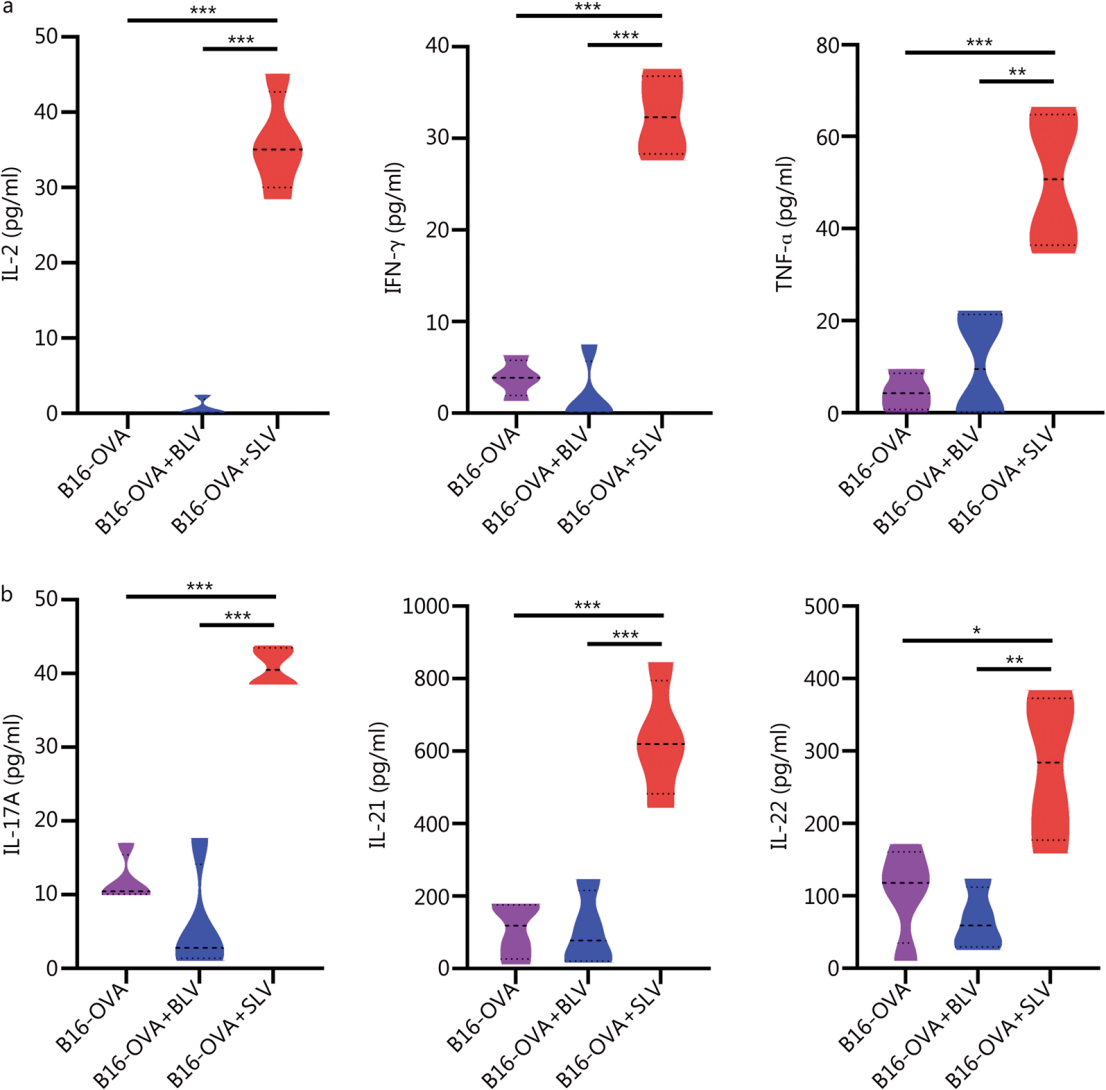
In situ host T-cell programming via PEGDA–PLL implants leads to Th1 and Th17-based responses. Quantification of Th1 cytokines (**a**) and Th17 cytokines (**b**) levels in serum analyzed using multiplexing. ^*^P < 0.05, ^**^P < 0.01, ^***^P < 0.001, one-way ANOVA with Tukey’s test. OVA ovalbumin, PEGDA–PLL polyethylene glycol diacrylate poly-L-lysine, BLV bolus lentiviruses, SLV scaffold loaded lentiviruses, IL-2 interleukin-2, IFN-γ interferon-γ, TNF-ɑ tumor necrosis factor-ɑ, IL-17A interleukin-17A, IL-21 interleukin-21, IL-22 interleukin 22

To investigate the infiltration of programmed host T-cells in solid tumor, the tumor tissue was extracted from mice on day 20 and cells were isolated for immunophenotyping (Additional file 1: Figs. S10, S11). A remarkably higher percentage of CD3^+^ cells [(19.6 ± 3.0)%] was observed in tumors isolated from mice implanted with SLV matrices (Fig. 7a). Moreover, elevated numbers of CD3^+^CD8^+^OVA-TCR^+^ (Fig. 7b), CD3^+^CD8^−^OVA-TCR^+^ (Additional file 1: Fig. S12a), CD3^+^CD4^+^ and CD3^+^CD8^+^ cells (Additional file 1: Fig. S12b) were detected in the SLV group compared to other control groups. This validates the tumor infiltration capability of in situ programmed host T-cells and corroborates that these cells were functional and produced a robust anti-tumor immune response. In addition, the CD4:CD8 T-cell ratio was significantly lower in the tumor due to increased number of CD8^+^ T-cells (Fig. 7c). More rigorous immunophenotyping of cells isolated from tumors revealed a significant reduction in the immunosuppressive cell population, namely, CD3^+^CD4^+^FOXP3^+^ Treg cells (Additional file 1: Fig. S12c) and F4/80^+^CD206^+^ M2 macrophages (Fig. 7d). We also observed an increased infiltration of other cellular phenotypes such as F4/80^+^CD80^+^ M1 macrophages (Fig. 7e), CD11c^+^ dendritic cells (Fig. 7f), CD14^+^ monocytes (Fig. 7g) and CD19^+^ B cells (Fig. 7h) in the tumor of SLV group when compared with other control conditions such as no treatment (B16-OVA) and BLV groups. Overall, the percentage of immune cells in the tumor increased significantly and correlated with reduction in tumor size. Th1 and Th17 cytokines also play an important and diverse role in a variety of solid tumors and their combinations have been shown to influence tumor progression. Therefore, we analyzed the cytokine levels in these experimental groups on day 15 and found that most cytokine levels were significantly increased (twofold or more). Th1 cytokines such as IL-2, IFN-γ and TNF-ɑ were higher in the SLV as compared to the BLV group and the no treatment control group (Fig. 8a). In addition, Th17 cytokines such as IL-17A, IL-21 and IL-22 were also found to increase under implantation conditions in comparison to other groups (Fig. 8b). Overall, (1) reduction in tumor size; (2) increased tumor-specific T-cells in tumor and other lymphoid organs; (3) elevated numbers of antigen-presenting cells; (4) reduction of immunosuppressive cells in tumor; and (5) elevated levels of Th1 and Th17-related cytokines in serum, demonstrate the anti-tumor efficacy of LV loaded PEGDA–PLL implant, suggesting the significance and possibility of further development of this strategy for treatment of solid tumors.

## Discussion

This study shows for the first time that 3D scaffolds loaded with LVs encoding tumor-specific receptor genes can be used to program host T-cells in situ and provide antigen-recognition to exert anti-tumor efficacy in solid tumors. Our approach differs from existing approaches for generation of tumor-specific T-cells, which require the isolation of patients’ T-cells and cumbersome protocols for their subsequent in vitro genetic modification by means of retroviral or lentiviral vectors, followed by infusion of programmed tumor-specific T-cells into the body. Our strategy simplifies the traditional and complex in vitro protocols required for adoptive T-cell therapy, to a one-step one-day process that involves surgical implantation of a bioactive scaffold in vivo. In comparison to the cellular approaches, which include delivery of T-cells via biomaterials [16, 17] or co-incubating LVs and host T-cells in a scaffold prior to their delivery [24], our strategy offers a major advance as an acellular strategy that overcomes the need for any in vitro cell culture protocol.

We have demonstrated that a 3D macroporous, biocompatible scaffold made of PEGDA after functionalization with PLL facilitates LV immobilization (Fig. 3a) and improves transduction of infiltrating T-cells (Fig. 6a) due to enhanced cell-virus interaction within the larger area provided by the macroporous network (Fig. 1c, Additional file 1: Fig. S1). The charge-based electrostatic polymer-receptor interactions in PEGDA–PLL, which are completely absent in PEGDA, also contribute to cell attachment, but to a lesser extent (Fig. 1f, Additional file 1: Fig. S1d). In addition, it is known that the inclusion of macropores within the scaffold while enhancing cellular infiltration also improves angiogenesis [25]. This approach for lentiviral immobilization is superior to systemic delivery of LVs (Fig. 3d–f) not only because it minimizes off-target immunogenicity but also serves as a local gene pool and potentially protects the virus from neutralization [39, 40]*.* The synergistic effect of T-cell activation coupled with highly efficient lentiviral transduction and subsequent chemokine production leading to the migration of transduced T-cells (Fig. 6d, g), results in a robust anti-tumor response (Fig. 5).

This strategy may overcome the potential limitations of conventional T-cell approach, including the loss of viability and terminal differentiation of infused T-cells that render them non-functional due to transfer from in vitro culture conditions to the dynamic microenvironment in vivo [12, 13]. Our strategy does not require the generation of large numbers of cells in existing cell-infusion methods and may partially overcome the cytokine release syndrome observed with these infusion based cellular strategies. Traditional in vitro T-cells strategies have achieved remarkable results in hematological malignancies, but their efficacy is limited due to the complexity and heterogeneity of solid tumors. This approach can be used in a variety of solid tumors, where treatment requires a local immune response. Depending on the type of tumor and its complexity, various immunomodulatory factors or adjuvants can be added to overcome the immunosuppression and heterogeneity in solid tumors, thereby synergistically enhancing the anti-tumor effects of the system. While we have demonstrated the potential of our scaffold-based strategy for solid tumor therapy in melanoma, it can be further investigated and developed to program cells in vivo for treatment of other pathophysiological conditions.

One of the drawbacks of our strategy is the use of a non-degradable polymer, PEGDA, to fabricate the cryogel implant. This choice was made to eliminate any potential variability, such as the loss of lentiviral content that may occur due to matrix breakdown. However, this PEGDA system can be chemically modified by introducing a readily degradable part, thus enabling temporally-controlled degradation of PEGDA over a defined span [47]. Furthermore, although our strategy enables genetic modification of T-cells, it is yet not specific. The non-CD3 cells were indeed transduced but did not show any response due to absence of co-stimulatory signals. This aspect can, however, be addressed by using a T-cell specific promoter downstream of the OVA-TCR plasmid to reduce any possible off-target effects of gene delivery. Moreover, clinical implementation of implanting of LV-loaded scaffolds will rely on procedure as well as the safety of LVs. The third generation of LVs has been considered safe and effective for clinical research [48]. However, it remains to be evaluated whether the benefits of in vivo genetic programming of T-cells outweigh the safety concerns of off-target gene delivery. Nevertheless, our scaffold-based acellular strategy for T-cell programming is a step forward as an alternative for T-cell based adoptive cell-therapies specifically for solid tumor immunotherapy.

## Conclusion

Taken together, the PEGDA–PLL implant serves as an all-in-one platform for transducing T-cells in vivo while allowing their subsequent mobilization, leading to a significant anti-tumor response. We developed a 3D scaffold loaded with LVs encoding tumor-specific receptor genes to program host T-cells in situ, thereby, bringing together key aspects of T-cells immuno-engineering on a single platform and shortening the entire process to one day. The 3D scaffold was fabricated using a non-immunogenic material to create a local niche for infiltrating host cells. The macroporosity of the scaffold facilitates LV immobilization and prevents off-target gene transfer that might result from its systemic delivery. Additionally, it provides an interface for interactions between viral particles and infiltrating T-cells. Our strategy provides a modular platform that can be applied to the design of other immune cells or to deliver other immunomodulatory factors that affect cellular functions. It also inspires new therapeutic approaches that may involve cell engineering for various therapeutic applications.

### Supplementary Information

**Additional file 1:** **Fig. S1** Characterization of matrices. **Fig. S2** In vivo cellular infiltration into the matrices. **Fig. S3** In vivo compatibility of matrices. **Fig. S4** Immobilization of lentiviruses (LVs) on matrices and in vitro transduction studies. **Fig. S5** Surgical implantation of matrices for anti-tumor studies. **Fig. S6** Effect of anti-tumor therapy on spleen. **Fig. S7** Gating strategy to characterize for transduced cells (OVA-TCR^+^) (**a)** and helper (CD4^+^) and cytotoxic (CD8^+^) T-cells (**b**) in spleen and inguinal lymph node of tumor bearing mice implanted with matrices loaded with OVA-TCR lentiviruses. **Fig. S8** Characterization of programmed cells in spleen and inguinal lymph node. **Fig. S9** Characterization of splenocytes for non-specific transduction. **Fig. S10** Phenotypic characterization of T-cells infiltrating the tumors. **Fig. S11** Phenotypic characterization of immune cells (other than T-cells) infiltrating the tumors. **Fig. S12** Quantification of cells infiltrating the tumors, representing percentage of CD3^+^CD8^−^(CD4^+^)OVA-TCR^+^ cells (**a**), CD3^+^CD4^+^ and CD3^+^CD8^+^ cells (**b**) and CD3^+^CD4^+^FOXP3^+^ Treg cells (**c**). **Table S1** Blood cell markers on day 3 of C57BL/6 mice implanted with PEGDA or PEGDA-PLL implants (*n* = 3).

## Data Availability

The data that support the findings of this study are available from the corresponding author upon reasonable request.

## Abbreviations

3D: Three dimensional
PLL: Poly-L-lysine
TCRs: T-cell receptors
OVA-TCR: Ovalbumin specific T-cell receptor
B16-OVA: B16F10 cells expressing ovalbumin
IL-2: Interleukin 2
PEGDA–PLL: Polyethylene glycol diacrylate poly-L-lysine
PEGDA: Polyethylene glycol
UV: Ultraviolet
EDC: 1-Ethyl-3-(3-dimethyl aminopropyl) carbodiimide
NHS: N-hydroxy-succinimide
PBS: Phosphate buffered saline
ASTM: American Society for Testing and Materials
MTT: 3-(4,5-Dimethythiazolyl-2)-2,5-diphenyltetrazolium bromide
CFSE: 5-(and 6)-carboxyfluorescein diacetate succinimidyl ester RPMI Roswell Park Memorial Institute
FBS: Fetal bovine serum
HEPES: 4-(2-Hydroxyethyl)-1-piperazineethanesulfonic acid
DMEM: Dulbecco’s modified Eagle’s medium
rhIL-2: Recombinant human IL-2
WT: Wild type
TNF-α: Tumor necrosis factor-α
EF1α: Elongation factor 1α
IFN-γ: Interferon-γ
SEM: Standard error mean
LVs: Lentiviruses
GFP LVs: Green fluorescent protein expressing lentiviruses
RFP: Red fluorescent protein
OVA-TCR LVs: Ovalbumin specific T-cell receptor expressing lentiviruses
CXCR: C-X-C motif chemokine receptor
CXCL: Chemokine C-X-C motif ligand
CCL: Chemokine ligand
