# Supplementary material for: Acellular scaffold-based approach for in situ genetic engineering of host T-cells in solid tumor immunotherapy
Source: Mil Med Res. 2024 Jan 4;11:3. doi: 10.1186/s40779-023-00503-6 (PMC10765574; doi:10.1186/s40779-023-00503-6)
Supplement: Supplementary file 1 — Additional file 1: Fig. S1 Characterization of matrices. Fig. S2 In vivo cellular infiltration into the matrices. Fig. S3 In vivo compatibility of matrices. Fig. S4 Immobilization of lentiviruses (LVs) on matrices and in vitro transduction studies. Fig. S5 Surgical implantation of matrices for anti-tumor studies. Fig. S6 Effect of anti-tumor therapy on spleen. Fig. S7 Gating strategy to characterize for transduced cells (OVA-TCR+) (a) and helper (CD4+) and cytotoxic (CD8+) T-cells (b) in spleen and inguinal lymph node of tumor bearing mice implanted with matrices loaded with OVA-TCR lentiviruses. Fig. S8 Characterization of programmed cells in spleen and inguinal lymph node. Fig. S9 Characterization of splenocytes for non-specific transduction. Fig. S10 Phenotypic characterization of T-cells infiltrating the tumors. Fig. S11 Phenotypic characterization of immune cells (other than T-cells) infiltrating the tumors. Fig. S12 Quantification of cells infiltrating the tumors, representing percentage of CD3+CD8−(CD4+)OVA-TCR+ cells (a), CD3+CD4+ and CD3+CD8+ cells (b) and CD3+CD4+FOXP3+ Treg cells (c). Table S1 Blood cell markers on day 3 of C57BL/6 mice implanted with PEGDA or PEGDA-PLL implants (n = 3). [file 40779_2023_503_MOESM1_ESM.docx]

**
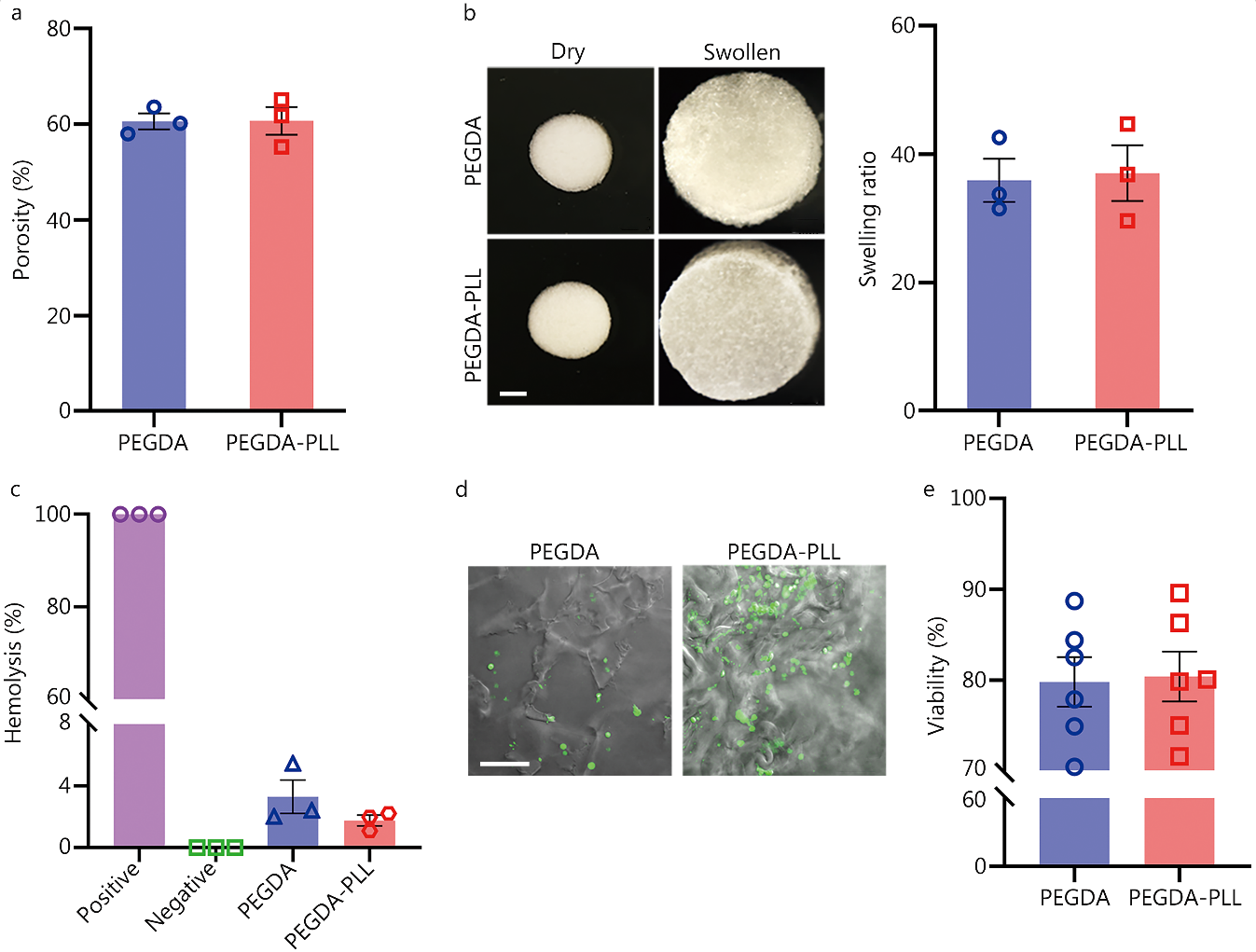
**

**Fig. S1** Characterization of matrices. **a** Porosity of PEGDA and PEGDA-PLL matrices. **b** Stereomicroscopy images of PEGDA and PEGDA-PLL matrices in dry and swollen states (top view), and quantified swelling ratio of PEGDA and PEGDA-PLL matrices. Scale bar = 2 mm. **c** Percentage of hemolysis when incubated with PEGDA and PEGDA-PLL matrices. Positive control represents complete hemolysis induced by lysis buffer and negative control represents no treatment of blood. **d** Confocal microscopy images of CFSE labeled T-cells cultured in PEGDA and PEGDA-PLL matrices. Scale bar = 100 µm. **e** Viability of T-cells cultured in matrices at 24 h. PEGDA polyethylene glycol diacrylate, PLL poly-L-lysine, CFSE 5-(and 6)-carboxyfluorescein diacetate succinimidyl ester


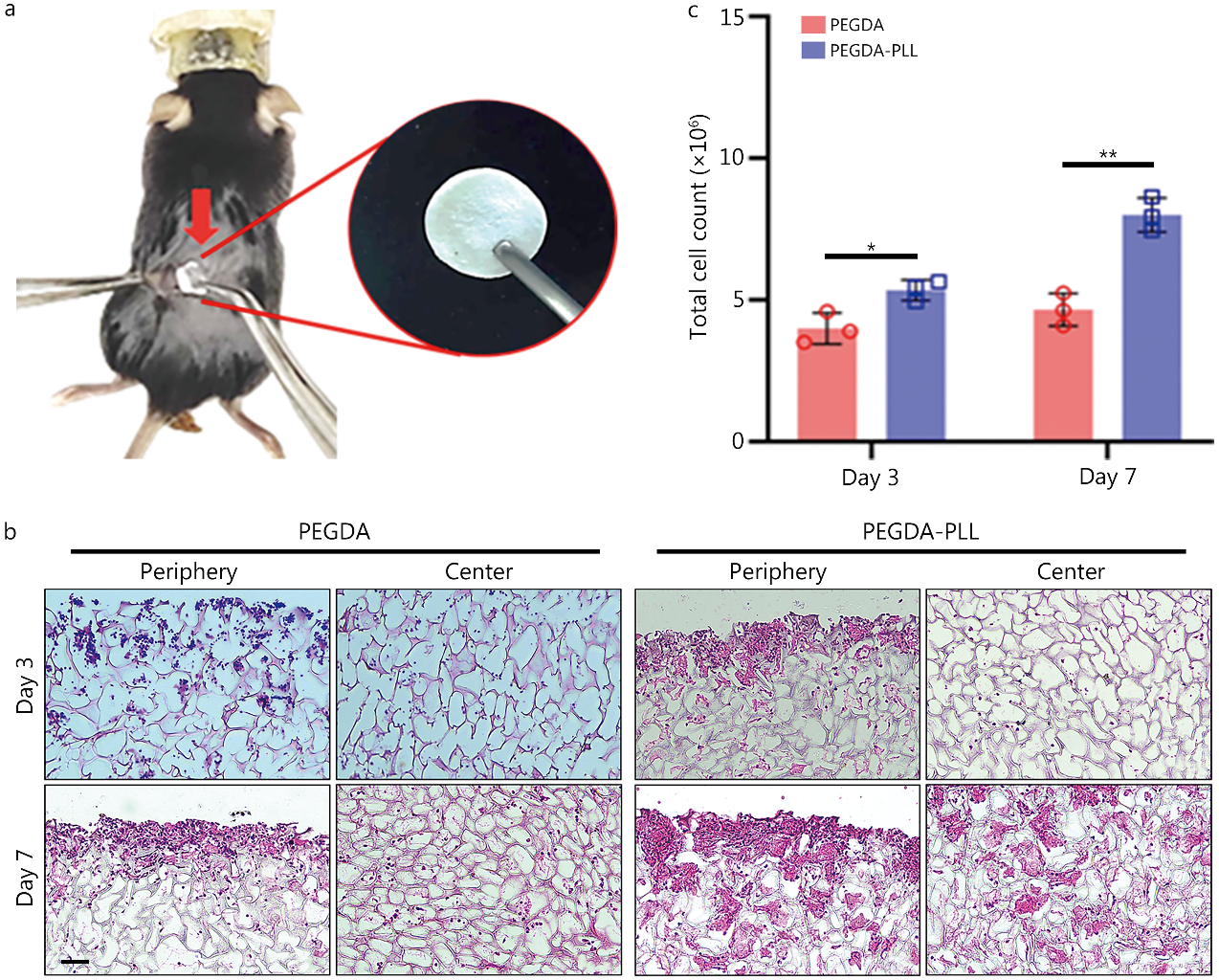


**Fig. S2** In vivo cellular infiltration into the matrices. **a** Photographs showing surgical procedure for implantation of PEGDA and PEGDA-PLL matrices in mice for in vivo studies. Red arrow indicates the site of implantation of the PEGDA or PEGDA-PLL matrices. **b** Representative images of HE-stained sections of matrices (periphery and center) when explanted at day 3 and day 7 after subcutaneous implantation in C57BL/6 immunocompetent mice. Scale bar = 100 µm. **c** Total viable count of cells infiltrated in PEGDA and PEGDA-PLL implant on day 3 and day 7. ^*^*P* < 0.05, ^**^*P* < 0.01, ^***^*P* < 0.001, one-way ANOVA with Tukey’s test. PEGDA polyethylene glycol diacrylate, PLL poly-L-lysine

**
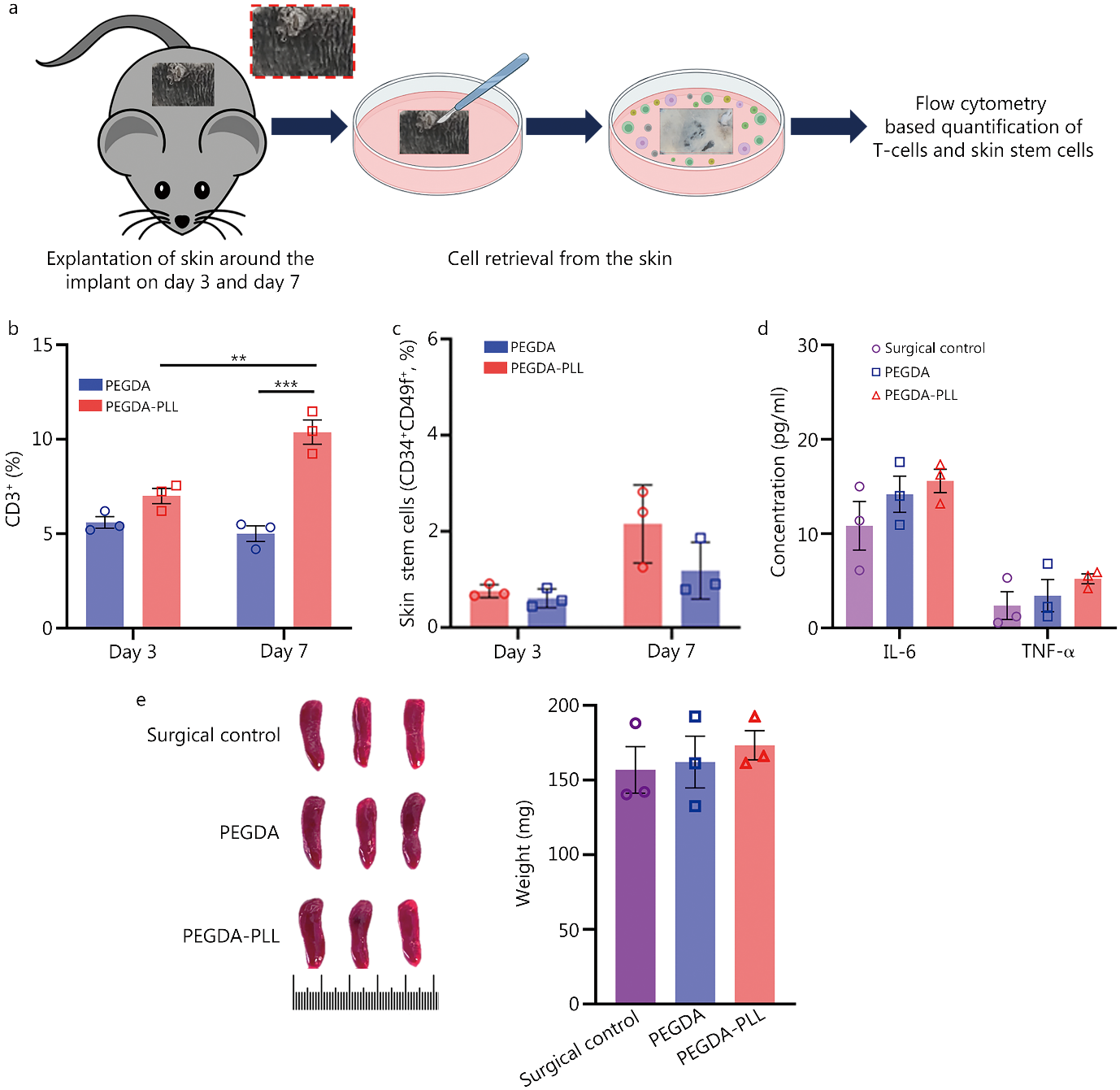
**

**Fig. S3** In vivo compatibility of matrices. **a** Experimental design for studying peripheral effect of the implant on skin. Quantification of skin resident T-cells (CD3^+^) (**b**) and skin stem cells (CD34^+^CD49f^+^) (**c**) in skin around the implanted PEGDA and PEGDA-PLL scaffolds removed at day 3 and day 7. **d** Concentrations of inflammatory cytokines (IL-6 and TNF-ɑ) measured in serum isolated from retro-orbitally collected blood, 7 d after implantation of PEGDA and PEGDA-PLL matrices. **e** Photographs and quantification of weights of spleens explanted on day 7 from mice with PEGDA or PEGDA-PLL matrices. Minimum unit of scale is 1 mm. Surgical control without any matrix was used to evaluate the inflammatory response to surgery. ^**^*P* < 0.01, ^***^*P* < 0.001, one-way ANOVA with Tukey’s test. PEGDA polyethylene glycol diacrylate, PLL poly-L-lysine, IL-6 interleukin-6, TNF-ɑ tumor necrosis factor-α


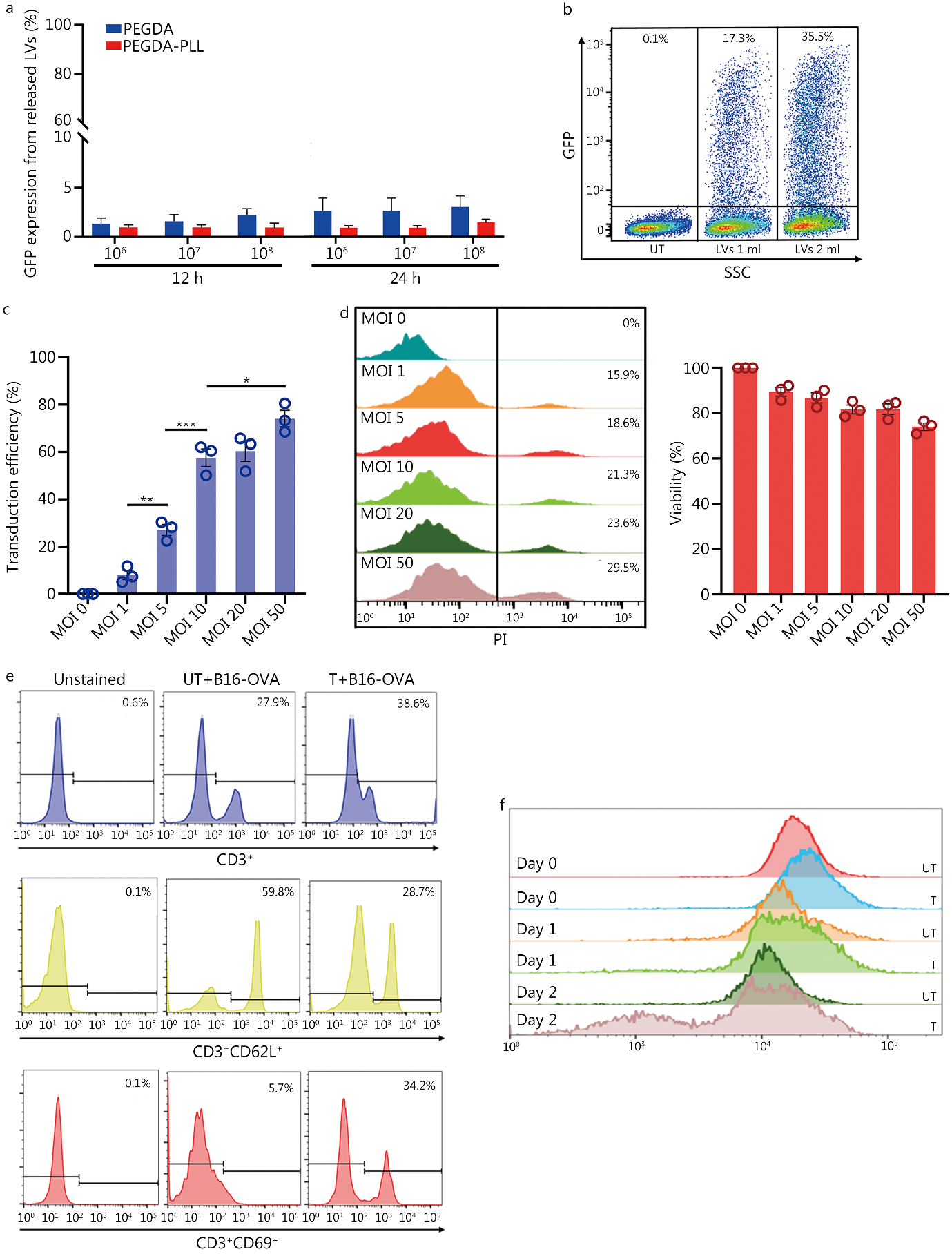

**Fig. S4** Immobilization of lentiviruses (LVs) on matrices and in vitro transduction studies. **a** Percent GFP expression in LentiX cells when incubated for 72 h with LVs released at 12 h and 24 h from matrices immobilized with varying numbers of lentiviral particles (10^6^, 10^7^, and 10^8^). **b** Flow cytometry dot plots showing GFP expression representing infectivity of LentiX cells from the supernatant (1 ml or 2 ml) of released LVs. **c** Bar graph showing transduction of LentiX cells with OVA-TCR encoding LVs at varying multiplicity of infection (MOI). **d** Overlaid flow cytometry histogram plots (left) and bar graphs quantifying the viability (right) of OVA-TCR expressing T-cells transduced with OVA-TCR encoding LVs at varying MOI. **e** Flow cytometry histogram plots showing expression of CD3 (T-cell marker), CD62L (naive T-cell marker) and CD69 (early activation marker) 48 h after co-culture of B16-OVA tumor cells with untransduced (UT) and transduced (T) OVA-TCR expressing T-cells. **f** CFSE proliferation on day 1 and day 2 of UT and T OVA-TCR expressing T-cells when co-cultured with B16-OVA tumor cells. PEGDA polyethylene glycol diacrylate, PLL poly-L-lysine, GFP green fluorescent protein, SSC side scatter, OVA-TCR ovalbumin T-cell receptor


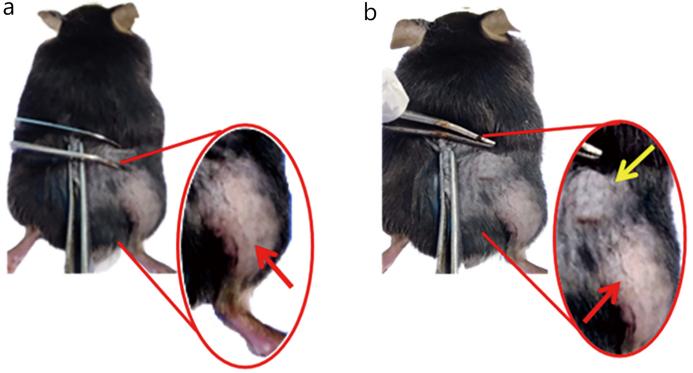


**Fig. S5** Surgical implantation of matrices for anti-tumor studies. **a** Mice showing palpable tumor and swollen mass of cell as indicated by red arrow on day 1. **b** Mice implanted with PEGDA-PLL cryogel (yellow arrow) near the site of tumor (red arrow) on day 1. PEGDA-PLL polyethylene glycol diacrylate poly-L-lysine


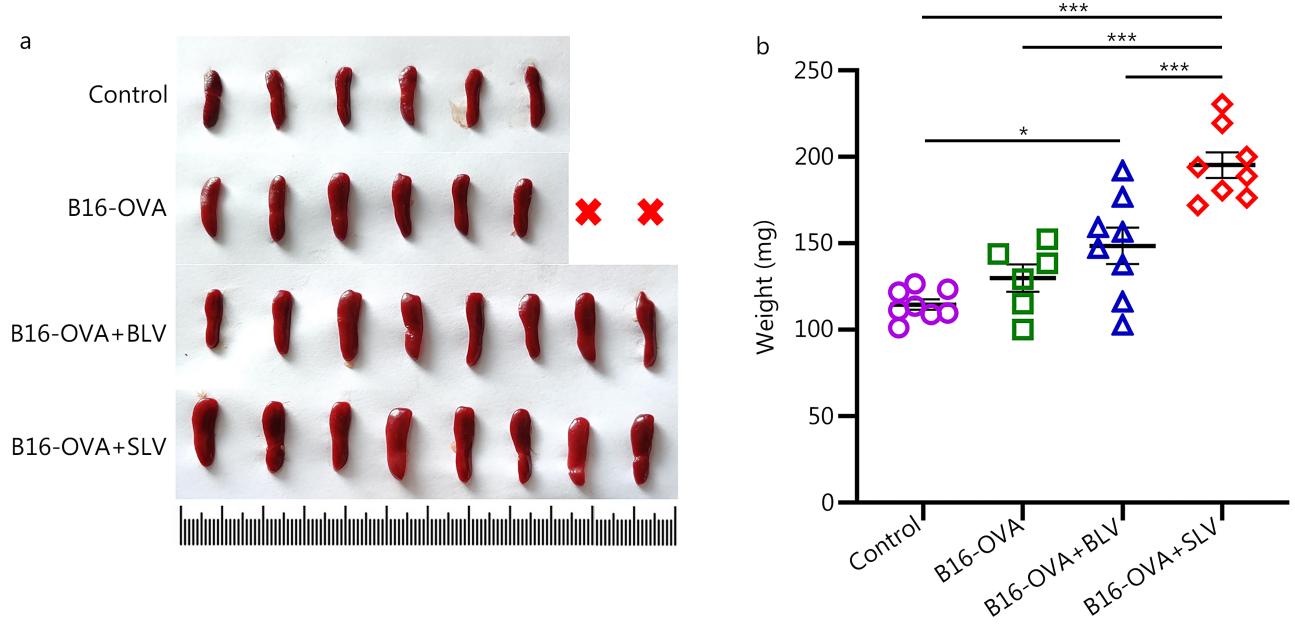


**Fig. S6** Effect of anti-tumor therapy on spleen. **a** Photographs of spleen from various experimental groups obtained after sacrificing the mice on day 20 for size comparison. Minimum unit of scale is 1 mm. **b** Weight of spleen for various experimental groups. Red cross represents the animal that died before the time point. ^*^*P* < 0.05, ^***^*P* < 0.001, one-way ANOVA with Tukey’s test. OVA ovalbumin, BLV bolus lentiviruses, SLV scaffold loaded lentiviruses


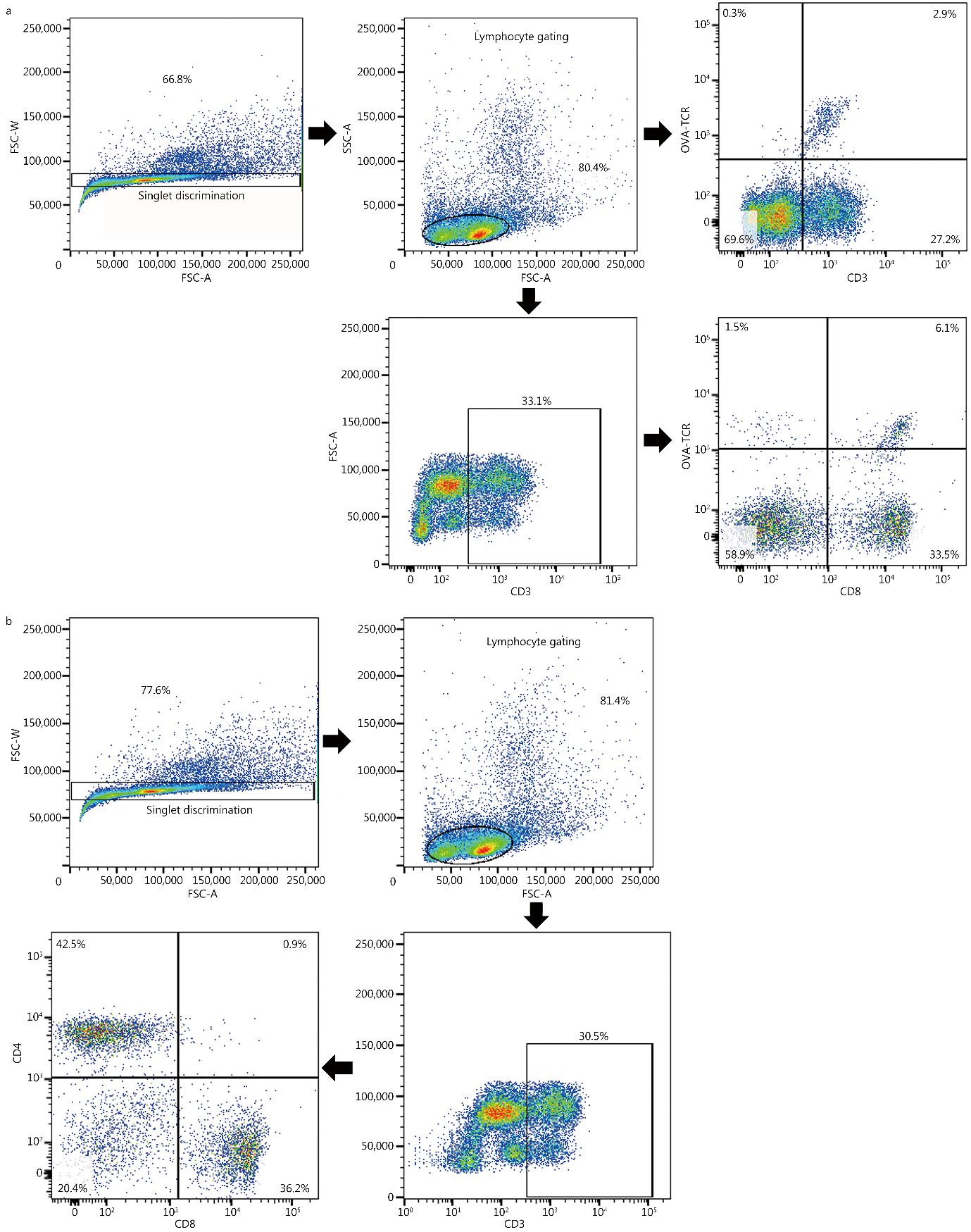


**Fig. S7** Gating strategy to characterize for transduced cells (OVA-TCR^+^) (**a)** and helper (CD4^+^) and cytotoxic (CD8^+^) T-cells (**b**) in spleen and inguinal lymph node of tumor bearing mice implanted with matrices loaded with OVA-TCR lentiviruses. FSC-W forward scatter width, FSC-A forward scatter area, SSC-A side scatter area, OVA-TCR ovalbumin T-cell receptor

**
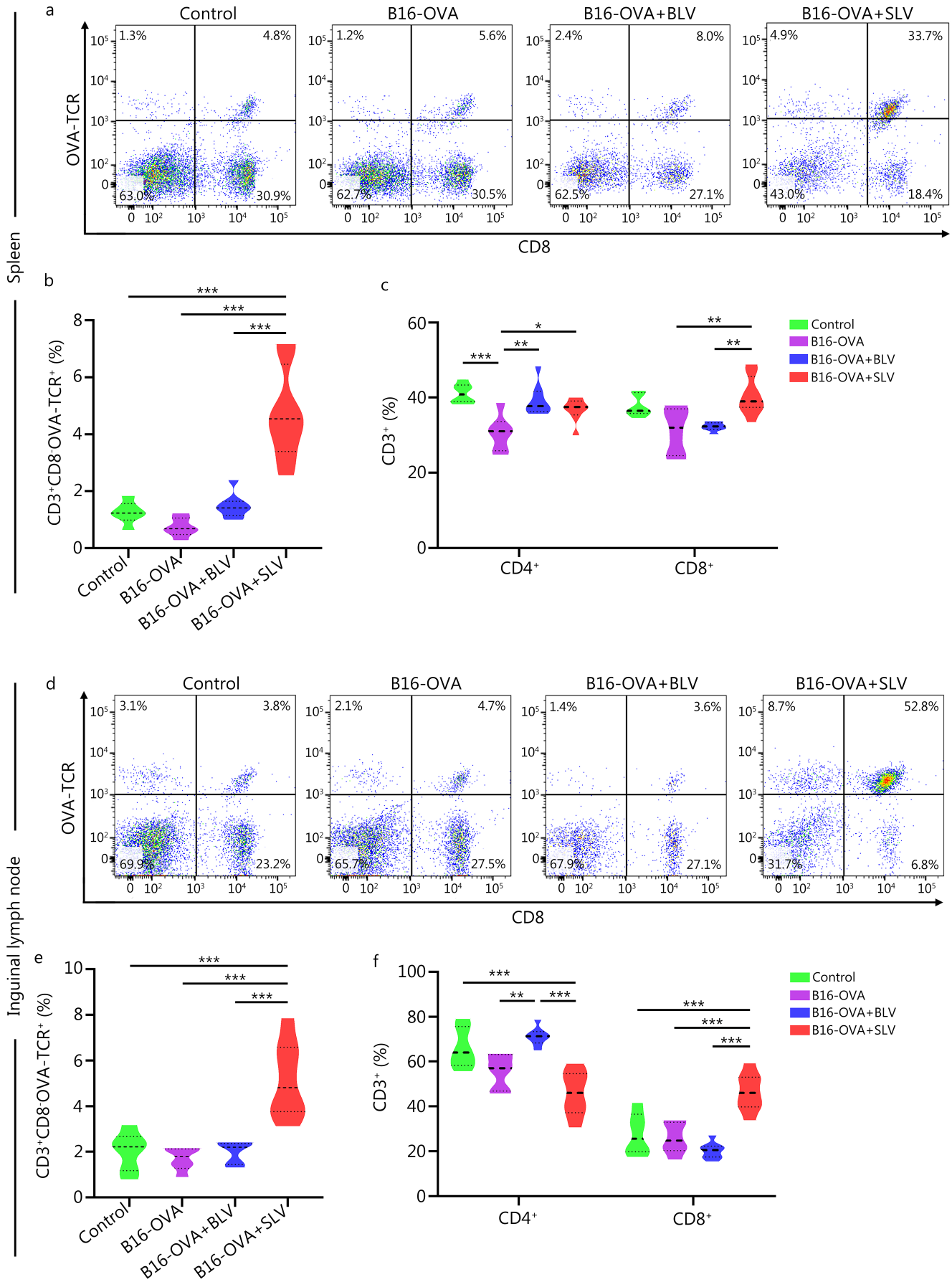
**

**Fig. S8** Characterization of programmed cells in spleen and inguinal lymph node. Flow cytometry based quantification showing in situ programming of T-cells quantified as percentage of OVA-TCR transduced cytotoxic (CD8^+^) T-cells (**a, d**), CD3^+^CD8^-^(CD4^+^)OVA-TCR^+^ cells (**b, e**), and CD3^+^CD4^+^ and CD3^+^CD8^+^ cells (**c, f**) in spleen (**a-c**) and inguinal lymph node (**d-f**) for different experimental groups, namely, Control (no tumor), B16-OVA (no treatment), B16-OVA + BLV (bolus LVs delivery), and B16-OVA + SLV (scaffold-mediated LVs delivery). ^*^*P* < 0.05, ^**^*P* < 0.01, ^***^*P* < 0.001, one-way ANOVA with Tukey’s test. OVA ovalbumin, TCR T-cell receptor, BLV bolus lentiviruses, SLV scaffold loaded lentiviruses


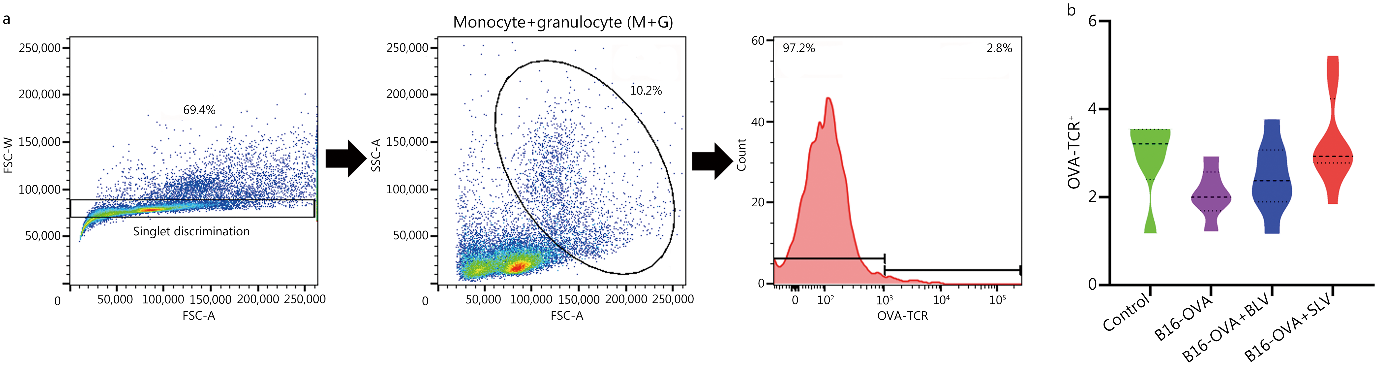


**Fig. S9** Characterization of splenocytes for non-specific transduction. **a** Gating strategy to analyse the non-specific transduction of cells (OVA-TCR^+^) in the monocyte-granulocyte population of spleen. **b** Quantification of OVA-TCR^+^ cells expressed as percentage of total monocyte-granulocyte population. FSC-W forward scatter width, FSC-A forward scatter area, SSC-A side scatter area, OVA-TCR ovalbumin T-cell receptor, BLV bolus lentiviruses, SLV scaffold loaded lentiviruses

**
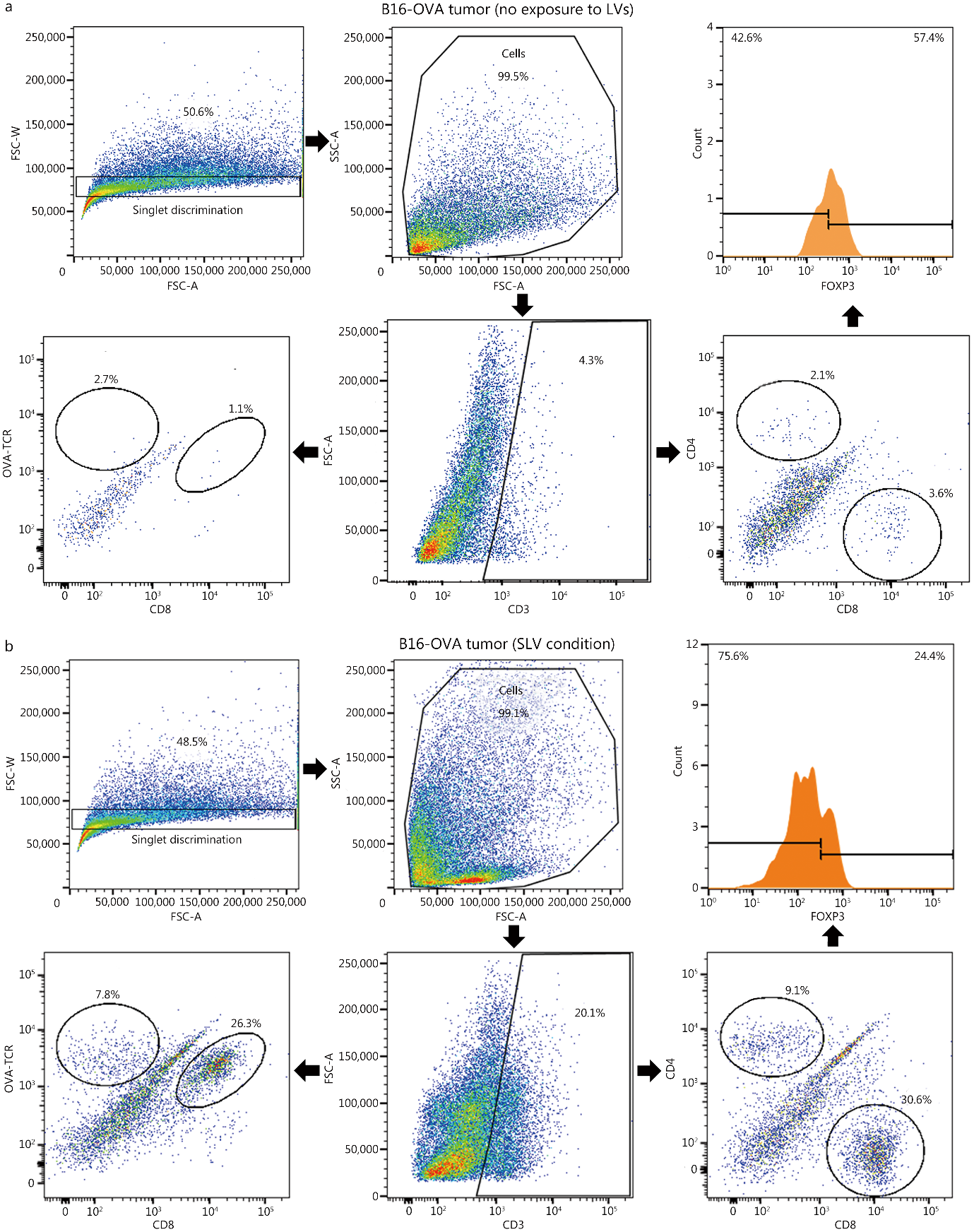
**

**Fig. S10** Phenotypic characterization of T-cells infiltrating the tumors. Gating strategy for T-cells infiltrating the tumors, namely, T-cells (CD3^+^), helper T-cells (CD3^+^CD4^+^), cytotoxic T-cells (CD3^+^CD8^+^), cytotoxic T-cells expressing OVA-TCR gene (CD3^+^CD8^+^OVA-TCR^+^), Treg cells (CD3^+^CD4^+^FOXP3^+^) for only B16-OVA (no exposure to LVs) condition (**a**) and B16-OVA+SLV condition (**b**). FSC-W forward scatter width, FSC-A forward scatter area, SSC-A side scatter area, OVA-TCR ovalbumin T-cell receptor, LVs lentiviruses, SLV scaffold loaded lentiviruses, FOXP3 forkhead box protein 3

**
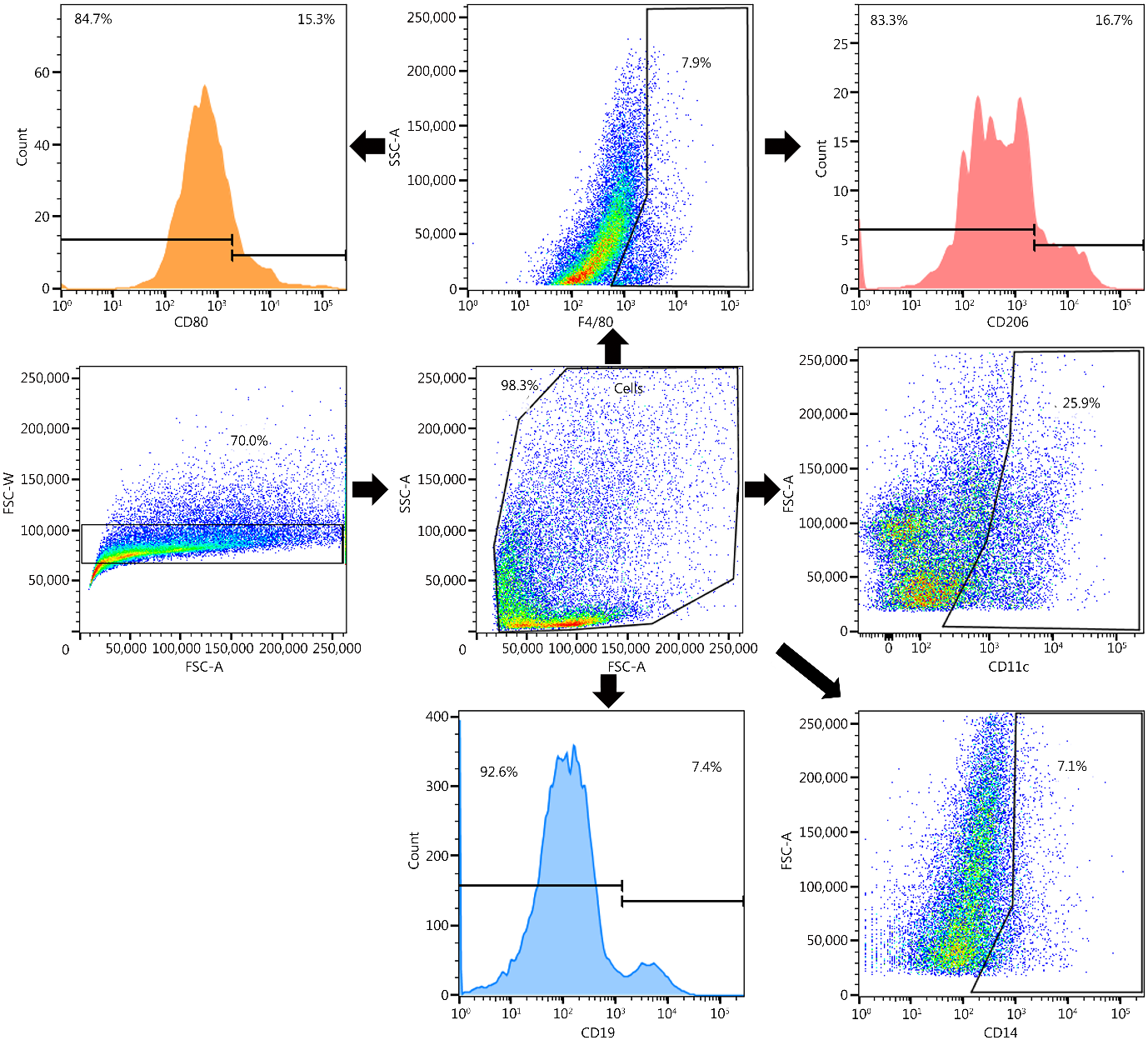
**

**Fig. S11** Phenotypic characterization of immune cells (other than T-cells) infiltrating the tumors. Gating strategy for M1 macrophages (F4/80^+^CD80^+^), M2 macrophages (F4/80^+^CD206^+^), dendritic cells (CD11c^+^), monocytes (CD14^+^) and B-cells (CD19^+^) infiltrating the tumors. FSC-W forward scatter width, FSC-A forward scatter area, SSC-A side scatter area


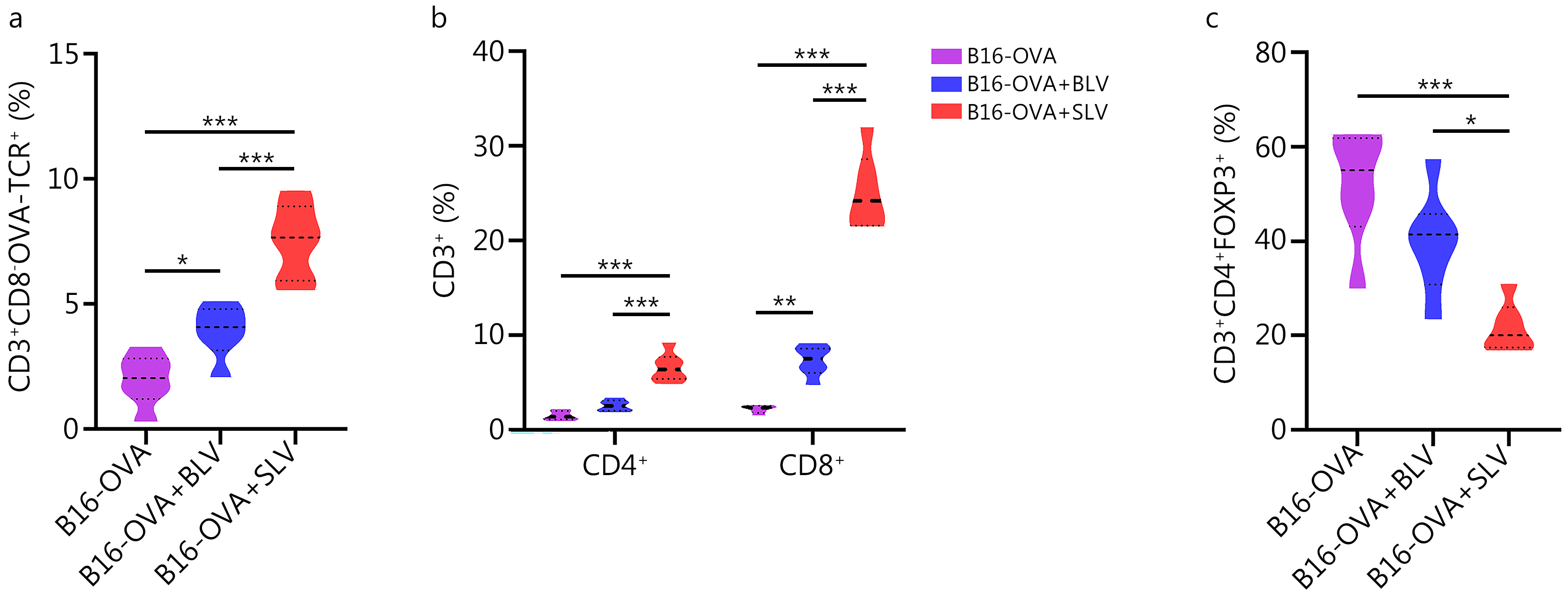


**Fig. S12** Quantification of cells infiltrating the tumors, representing percentage of CD3^+^CD8^-^(CD4^+^)OVA-TCR^+^ cells (**a**), CD3^+^CD4^+^ and CD3^+^CD8^+^ cells (**b**) and CD3^+^CD4^+^FOXP3^+^ Treg cells (**c**). ^*^*P* < 0.05, ^**^*P* < 0.01, ^***^*P* < 0.001, one-way ANOVA with Tukey’s test. OVA-TCR ovalbumin T-cell receptor, BLV bolus lentiviruses, SLV scaffold loaded lentiviruses, FOXP3 forkhead box protein 3

**Table S1** Blood cell markers in C57BL/6 mice on day 3 after PEGDA or PEGDA-PLL implantation (*n* = 3)

| **Blood cell markers** | **Surgical control** | **PEGDA** | **PEGDA-PLL** |
| --- | --- | --- | --- |
| WBC (× 10^3^/µl) | 3.96 ± 0.73 | 3.96 ± 1.62 | 3.96 ± 0.59 |
| LYM (× 10^3^/µl) | 2.49 ± 0.29 | 2.66 ± 0.96 | 2.68 ± 0.57 |
| MID (× 10^3^/µl) | 0.18 ± 0.01 | 0.18 ± 0.12 | 0.18 ± 0.05 |
| GRA (× 10^3^/µl) | 1.06 ± 0.08 | 1.38 ± 0.35 | 1.17 ± 0.07 |
| LYM% (%) | 66.90 ± 3.12 | 63.63 ± 2.59 | 67.60 ± 5.98 |
| MID% (%) | 4.43 ± 0.32 | 3.73 ± 2.01 | 4.67 ± 0.21 |
| GRA% (%) | 28.67 ± 2.92 | 34.13 ± 4.09 | 28.03 ± 5.86 |
| RBC (× 10^9^/ml) | 6.25 ± 0.15 | 5.73 ± 0.42 | 5.76 ± 0.26 |
| HGB (g/dl) | 10.03 ± 0.32 | 9.27 ± 0.51 | 9.17 ± 0.42 |
| MCHC (g/dl) | 338.97 ± 10.07 | 323.43 ± 9.46 | 320.07 ± 7.61 |
| MCH (pg) | 13.00 ± 6.07 | 16.23 ± 0.35 | 15.90 ± 0.44 |
| MCV (fL) | 48.20 ± 0.56 | 50.83 ± 1.37 | 49.73 ± 2.38 |
| RDWCU (%) | 12.93 ± 0.31 | 13.10 ± 0.10 | 12.93 ± 0.21 |
| RDWSD (fL) | 29.37 ± 0.97 | 30.97 ± 0.65 | 30.83 ± 1.80 |
| HCT (%) | 32.17 ± 3.51 | 29.00 ± 0.80 | 28.27 ± 1.29 |
| PLT (× 10^9^/L) | 621.33 ± 156.11 | 632.67 ± 117.37 | 719.00 ± 109.58 |
| MPV (fL) | 6.50 ± 0.26 | 6.67 ± 0.31 | 6.07 ± 0.21 |
| PDW (fL) | 12.13 ± 1.27 | 11.90 ± 1.59 | 10.77 ± 1.31 |
| PCT (%) | 0.40 ± 0.08 | 0.42 ± 0.06 | 0.44 ± 0.05 |
| P-LCR (%) | 4.00 ± 0.96 | 4.50 ± 1.18 | 2.63 ± 0.40 |

*PEGDA* polyethylene glycol diacrylate, *PLL* poly-L-lysine, *WBC* white blood cells, *LYM* lymphocytes, *MID* mid-range absolute count, *GRA* granulocytes, *RBC* red blood cells, *HGB* hemoglobin, *MCHC* mean corpuscular hemoglobin concentration, *MCH* mean corpuscular hemoglobin, *MCV* mean corpuscular volume, *RDWCU* red cell distribution width*, RDWSD* red cell distribution width standard deviation, *HCT* hematocrit test, *PLT* platelet, *MPV* mean platelet volume, *PDW* platelet distribution width, *PCT* procalcitonin, *P-LCR* platelet larger cell ratio
